# Intracellular zinc protects tumours from T cell-mediated cytotoxicity

**DOI:** 10.1038/s41418-024-01369-4

**Published:** 2024-09-11

**Authors:** Emily J. Lelliott, Jonathan Naddaf, Katherine Ganio, Jessica Michie, Shelly Wang, Lin Liu, Natasha Silke, Antonio Ahn, Kelly M. Ramsbottom, Amelia J. Brennan, Andrew J. Freeman, Shom Goel, Stephin J. Vervoort, Conor J. Kearney, Paul A. Beavis, Christopher A. McDevitt, John Silke, Jane Oliaro

**Affiliations:** 1 Cancer Research Division, Peter MacCallum Cancer Centre, Melbourne, VIC 3000 Australia; 2 Sir Peter MacCallum Department of Oncology, The University of Melbourne, Parkville, VIC 3010 Australia; 3 Department of Microbiology and Immunology, The Peter Doherty Institute for Infection and Immunity, The University of Melbourne, Parkville, VIC 3010 Australia; 4 Inflammation Division, The Walter and Eliza Hall Institute of Medical Research, Parkville, VIC 3052 Australia; 5 Department of Medical Biology, The University of Melbourne, Parkville, VIC 3010 Australia; 6 Present Address: Olivia Newton-John Cancer Research Institute, Heidelberg, VIC 3084 Australia

**Keywords:** Cell death and immune response, Immune evasion

## Abstract

Tumour immune evasion presents a significant challenge to the effectiveness of cancer immunotherapies. Recent advances in high-throughput screening techniques have uncovered that loss of antigen presentation and cytokine signalling pathways are central mechanisms by which tumours evade T cell immunity. To uncover additional vulnerabilities in tumour cells beyond the well-recognized antigen presentation pathway, we conducted a genome-wide CRISPR/Cas9 screen to identify genes that mediate resistance to chimeric-antigen receptor (CAR)-T cells, which function independently of classical antigen presentation. Our study revealed that loss of core-binding factor subunit beta (CBFβ) enhances tumour cell resistance to T cell killing, mediated through T cell-derived TNF. Mechanistically, RNA-sequencing and elemental analyses revealed that deletion of CBFβ disrupts numerous pathways including those involved in zinc homoeostasis. Moreover, we demonstrated that modulation of cellular zinc, achieved by supplementation or chelation, significantly altered tumour cell susceptibility to TNF by regulating the levels of inhibitor of apoptosis proteins. Consistent with this, treatment of tumour cells with a membrane-permeable zinc chelator had no impact on tumour cell viability alone, but significantly increased tumour cell lysis by CD8+ T cells in a TNF-dependent but perforin-independent manner. These results underscore the crucial role of intracellular zinc in regulating tumour cell susceptibility to T cell-mediated killing, revealing a novel vulnerability in tumour cells that might be exploited for the development of future cancer immunotherapeutics.

## Introduction

The immune system plays a critical role in controlling tumour cell growth and survival [1]. Cytotoxic T cells can directly kill cancer cells with remarkable efficacy, and harnessing this activity is a central goal of the most widely used cancer immunotherapies; immune checkpoint inhibition (ICI) and chimeric antigen receptor (CAR)-T cell therapy [2]. However, while these immunotherapies can be highly effective in some cancers, the survival benefit from these treatments is limited to a subset of patients. It is therefore crucial to understand the mechanisms by which tumours evade T cell immunity in order to enhance existing immunotherapies and develop new T cell-directed immunotherapies for cancer patients.

For T cells to carry out their anti-tumour function, they need to recognise their cognate antigen presented on major histocompatibility (MHC) molecules on the tumour cell surface. Once the target antigen is recognized, T cells elicit anti-tumour effects through two primary mechanisms. First, they form an immune synapse with the tumour cell, leading to the secretion of cytotoxic molecules, including perforin and granzymes, that induce tumour cell death via the intrinsic apoptotic pathway [3, 4]. Second, T cells produce cytokines, such as tumour necrosis factor (TNF), that can trigger tumour cell death via the extrinsic apoptotic pathway [5, 6]. Indeed, previous studies applying genome-wide genetic screens in tumour cells have consistently identified that a loss of genes in either antigen presentation or cytokine pathways promotes tumour cell resistance to T cell killing [5, 7].

In this study, we aimed to identify tumour cell mechanisms of T cell evasion beyond well-known pathways involved in MHC-restricted antigen presentation, with the goal of uncovering novel vulnerabilities in tumour cells that could be targets for therapeutic intervention. To achieve this, a comprehensive whole-genome loss-of-function screen was conducted to identify genes that mediate tumour cell resistance to killing by CAR-T cells, as CAR-mediated T cell activation is not restricted to tumour antigen presentation on MHC molecules. Using this approach, coupled with RNA-sequencing analyses across different tumour lines, we identified intracellular zinc homoeostasis as critical for tumour cell susceptibility to T cell killing. Specifically, altering zinc levels influenced the sensitivity of tumour cells to T cell-derived TNF, a potent cytokine involved in tumour cell elimination. These findings suggest that modulating intracellular zinc levels could be a potential strategy to enhance the susceptibility of tumour cells to T cell killing mediated through TNF. Tumour intracellular zinc homoeostasis thus presents a novel target for the design of new therapeutic approaches that may overcome tumour immune evasion and enhance the efficacy of cancer immunotherapies.

## Results

### Genome-wide CRISPR/Cas9 screen identifies CBFβ as a regulator of tumour cell sensitivity to T cell-derived TNF

**Fig. 1:**
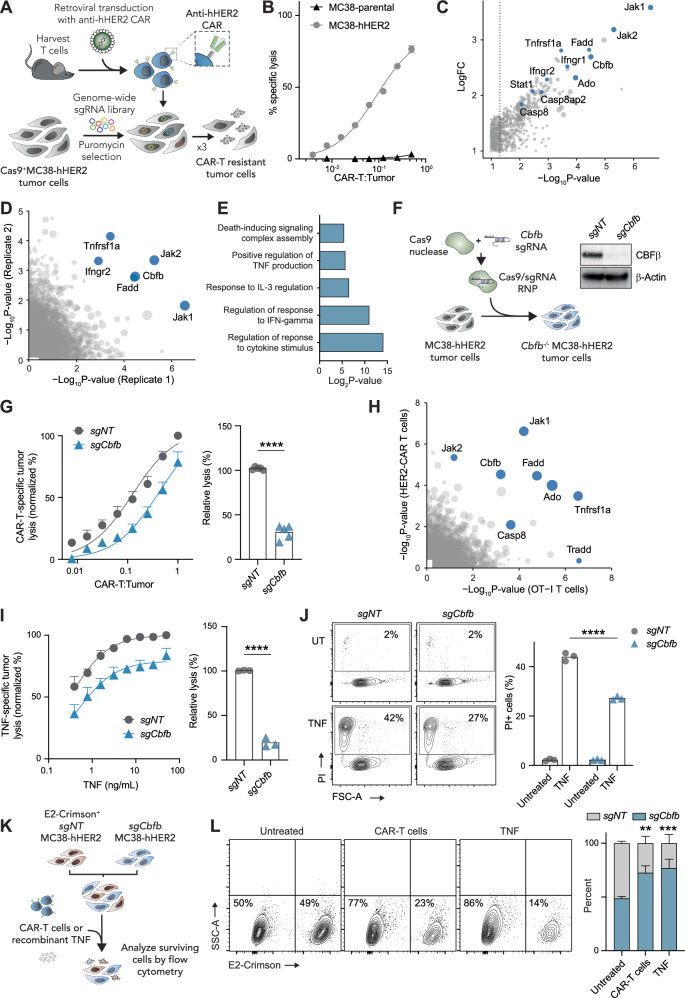
Genome-wide CRISPR/Cas9 screen identifies *Cbfb* as a regulator of tumour sensitivity to T cell derived TNF. **A** Schematic of CAR-T cell CRISPR/Cas9 screen. T cells from C57BL/6 mice were activated and retrovirally transduced to express CARs targeting hHER2 and cultured with MC38 tumour cells engineered to express hHER2, Cas9 and a genome-wide sgRNA library. Enriched guides were sequenced following 3 rounds of selection. **B** Lysis of ^51^Cr-labelled parental or hHER2-expressing MC38 tumour cells by hHER2-directed CAR-T cells in an 18 h co-culture, measured by ^51^Cr at increasing CAR-T to tumour cell ratios, representative plot of *n* = 3. **C**, **D** Enriched sgRNA from screen in **A**. **C** Representative plot of duplicate screens. **D** Shared sgRNA enrichment from each replicate screen. **E** GO term analysis showing enriched biological processes for top screen hits. **F** Schematic of CRISPR/Cas9-mediated genetic deletion of *Cbfb* in tumour cells by electroporation with ribonucleoprotein of Cas9 nuclease complexed to sgRNA targeting *Cbfb (sgCbfb)*, or a non-targeting control (*sgNT*), and immunoblot validation of CBFβ deletion in MC38 cells. **G** Lysis of ^51^Cr-labelled MC38-hHER2 tumour cells by hHER2-directed CAR-T cells in a 16 h co-culture, measured by ^51^Cr release at increasing CAR-T to tumour cell ratios. Relative lysis is calculated as the efficiency of CAR-T cells to achieve an equal percent lysis of tumour cells, unpaired t-test, *n* = 5. **H** Shared sgRNA enrichment from screen in **A** and a screen for MC38-OVA resistance to OT-I T cell killing [5]. **I** Lysis of ^51^Cr-labelled MC38 tumour cells in increasing concentrations of TNF over 16 h. Relative lysis is calculated as the efficiency of TNF to achieve an equal percent lysis of tumour cells, unpaired t test, *n* = 3. **J** Representative flow cytometry plots of MC38 tumour cells with death measured by PI uptake following 16 h of treatment with 10 ng/mL TNF (left), *n* = 4 quantified 2way ANOVA. **K** Schematic of competition assay to assess relative resistance of MC38 cells to CAR-T cells or TNF. MC38-hHER2*-sgNT* control cells were labelled with E2-Crimson and co-cultured with unlabelled MC38-hHER2-*sgCbfb* cells, followed by the addition of CAR-T cells (at a CAR-T to tumour cell ration of 2:1) or 10 ng/mL TNF for 16 h. **L** Representative flow cytometry plots (left) and *n* = 3 quantification of competition assay (right). All error bars show +/−SEM, ***P < 0.001, ****P < 0.0001.

To identify genes involved in tumour cell resistance to T cell killing independent of antigen presentation on MHC, we performed an unbiased whole-genome loss-of-function CRISPR/Cas9 screen in vitro, utilising HER2-directed second-generation mouse CAR-T cells (Fig. 1A). In this system, CAR-T cells recognise tumour cells engineered to express truncated human HER2 and induce tumour cell lysis in a HER2-specific manner (Fig. 1B). To carry out the screen, we transduced Cas9 + MC38-HER2 cells (a mouse colon cancer cell line) with a whole-genome single-guide RNA (sgRNA) library and subjected the cells to three successive rounds of overnight co-culture with CAR-T cells (Fig. 1A). The resulting population of cells was sequenced to detect enriched sgRNA and identify genes that promote tumour cell susceptibility to killing by CAR-T cells. Consistent with previous reports [5–7], we found enrichment of sgRNA targeting genes associated with both IFN-gamma signalling (*Jak1*, *Jak2*, *Ifngr2*) and TNF signalling (*Tnfrsf1a*, *Fadd*) in tumour cells resistant to CAR-T cell killing (Fig. 1C). This result was highly reproducible between two replicate screens (Fig. 1D). Further Gene Ontology (GO) term analyses confirmed that genes involved in the regulation of response to cytokine stimulus were the most significantly enriched (Fig. 1E). Together these results indicated that tumour cell resistance to CAR-T cells was predominately mediated through a loss of cytokine signalling.

In addition to previously identified genes involved in cytokine responses [5], we identified the gene *Cbfβ* among our most significantly enriched sgRNA. To validate that loss of CBFβ confers tumour cell resistance to CAR-T cell killing, we depleted CBFβ in MC38-HER2 tumour cells by electroporating cells with a CRISPR/Cas9-sgCbfb ribonucleoprotein (RNP) complex (Fig. 1F). We then assessed the sensitivity of CBFβ -depleted cells (sgCbfb) to killing by CAR-T cells, compared to control tumour cells electroporated with a non-targeting sgRNA (sgNT). Consistent with our screen results, we found that sgCbfb cells were significantly more resistant to CAR-T cell killing compared to the sgNT control cells (Fig. 1G).

To determine if CBFβ presents a tumour cell vulnerability specific to CAR-T cells, or is more broadly involved in the regulation of CD8+ T cell evasion, we next compared the enriched sgRNA from our CAR-T cell screen with the enriched sgRNA from our previously published OT-I T cell resistance screen in MC38-OVA cells [5] (Fig. 1H). As expected, the most significantly enriched overlapping sgRNA between these screens targeted genes involved in cytokine signalling. *Cbfβ* was also among the most significantly enriched overlapping genes, suggesting that loss of CBFβ promotes tumour cell resistance to T cell killing through a mechanism common to both CAR- and TCR-mediated tumour cell recognition.

Given the strong enrichment of sgRNA targeting genes associated with cytokine signalling in our screen, we examined whether loss of CBFβ modulated tumour sensitivity to TNF; a key cytokine known to induce tumour cell death [5, 6]. To interrogate this, we measured cell death following exposure to increasing concentrations of recombinant TNF. Consistent with our CAR-T cell results, we found that sgCbfb cells were significantly more resistant to TNF-mediated cell death than sgNT control cells (Fig. 1I, J). To further confirm a role for CBFβ in modulating tumour cell sensitivity to both CAR-T cell and TNF-mediated death, we carried out competitive cell death assays by mixing E2-Crimson-labelled sgNT cells with unlabelled sgCbfb cells a 1:1 ratio and exposed the mixed population to CAR-T cells, or recombinant TNF (Fig. 1K). Analyses of the surviving tumour cell population by flow cytometry confirmed significant enrichment of the CBFβ-depleted tumour cells under both conditions (Fig. 1L), further indicating that CBFβ regulates tumour cell sensitivity to T cell-derived TNF.

### Loss of CBFβ disrupts tumour intracellular metal ion homoeostasis

**Fig. 2:**
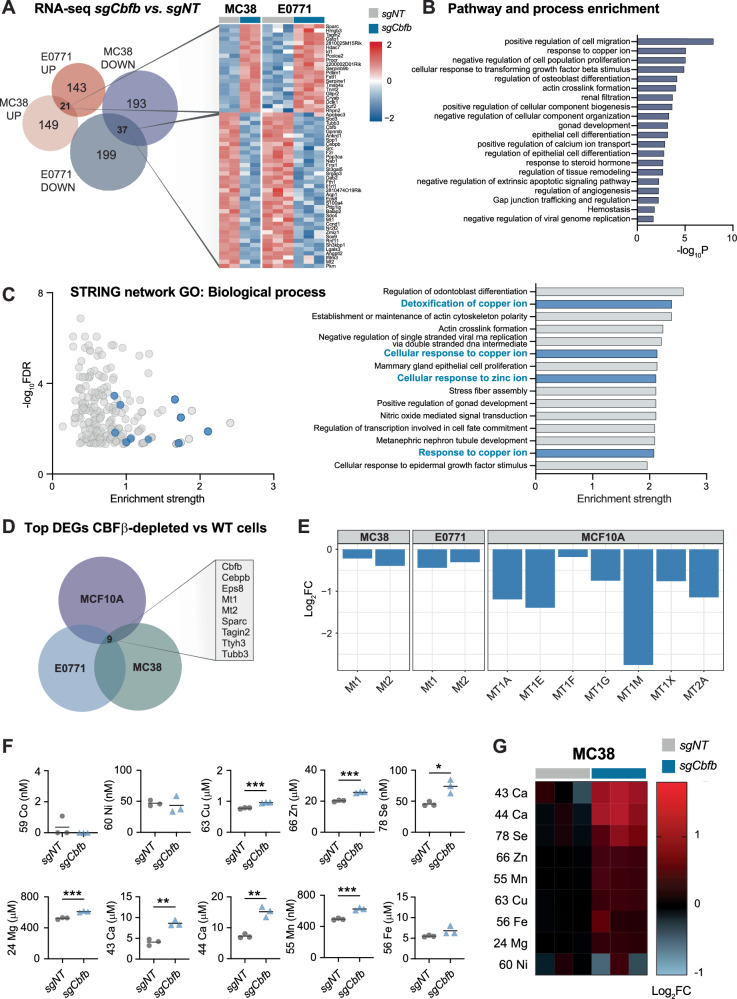
Loss of *Cbfb* disrupts tumour intracellular metal ion homoeostasis. **A**–**E** RNA-seq on wild-type and CBFβ-depleted tumour cells. **A** Top 400 differentially expressed genes (DEGs) significantly up- or down-regulated in *sgCbfb* versus *sgNT* MC38 and E0771 tumour cells. Heatmap shows common genes differentially expressed in both cell lines. **B** Pathway and process enrichment analysis on common DEGs from **A** using Metascape. **C** STRING network analysis on common DEGs showing enriched GO biological processes; pathways relating to metal ion homoeostasis are shown in blue. **D** Top 400 significant DEGs in *sgCbfb* versus *sgNT* MC38 and E0771 tumour cells, and CBFB KO vs. WT human breast cancer MCF10A tumour cells [8], showing common DEGs across all three cell lines. **E** Differential expression of metallothionein genes in CBFβ-depleted vs WT tumour cells. **F**, **G** Intracellular metal ion concentration in *sgNT* and *sgCbfb* MC38 tumour cells, measured by ICP-MS, unpaired t-test, *n* = 3. **G** Differential intracellular metal ion concentration measured by ICP-MS in *sgCbfb* vs. *sgNT* MC38 tumour cells, columns represent replicate samples. *P < 0.05, **P < 0.01, ***P < 0.001.

To explore the mechanism by which deletion of CBFβ modulates tumour cell sensitivity to TNF, we compared the transcriptional profile of sgNT and sgCbfb cells by bulk RNA-sequencing (RNA-seq). To narrow down conserved differential transcripts resulting from the deletion of CBFβ, we performed RNA-seq analyses in both MC38 cells, and a second cancer cell line, E0771 (mouse breast cancer), and used Venn analysis to identify common differentially expressed genes (DEGs) between sgCbfb and sgNT cells across both cancer lines (Fig. 2A). Consistent with our results in MC38 cells, deletion of CBFβ in E0771 cells promoted resistance to TNF-mediated death (Supplementary Fig. S1). Among the top 400 DEGs in each cancer line, we identified 21 commonly upregulated genes and 37 commonly downregulated genes in sgCbfb cells versus the sgNT control cells (Fig. 2A). Further investigation of this conserved differential gene set by pathway and network analyses revealed strong enrichment of processes associated with intracellular metal ion homoeostasis, including cellular responses to copper and zinc (Fig. 2B, C).

We next investigated the applicability of our results to human cancer by comparing our DEG analyses in mouse MC38 and E0771 cancer cells to a published RNA-seq dataset from wildtype and CBFβ deficient human breast cancer cells (MCF10A) [8]. Again, using Venn analysis on the top 400 DEGs from each dataset, we identified 9 common DEGs across mouse and human cancer models (Fig. 2D). Among these were genes encoding metallothionines (Mt), which are small, highly conserved metal-binding proteins that function in the homoeostatic maintenance of intracellular copper, zinc, and other metal ions [9, 10]. Across all cancer models, we found several Mt genes downregulated in CBFβ-deficient cells, including Mt1 and Mt2 (mouse) and MT1A, MT1E, MT1F, MT1G, MT1M, MT1X, and MT2A (human) (Fig. 2E), further suggesting that metal ion homoeostasis is substantially altered in these cells. To directly examine the effects of CBFβ deletion on cellular metal homoeostasis, inductively coupled plasma-mass spectrometry (ICP-MS) was used to quantitatively assess the elemental profiles of MC38 sgCbfb cells and sgNT control cells. Consistent with our transcriptomic data, we found that deletion of CBFβ was associated with a significant increase in the total cellular concentration of almost all elements, including the Mt-interacting metals copper and zinc (Fig. 2F, G). Taken together, these data show that loss of CBFβ disrupts the homoeostasis of cellular metal ions.

### Zinc promotes tumour cell resistance to TNF-mediated cell death

**Fig. 3:**
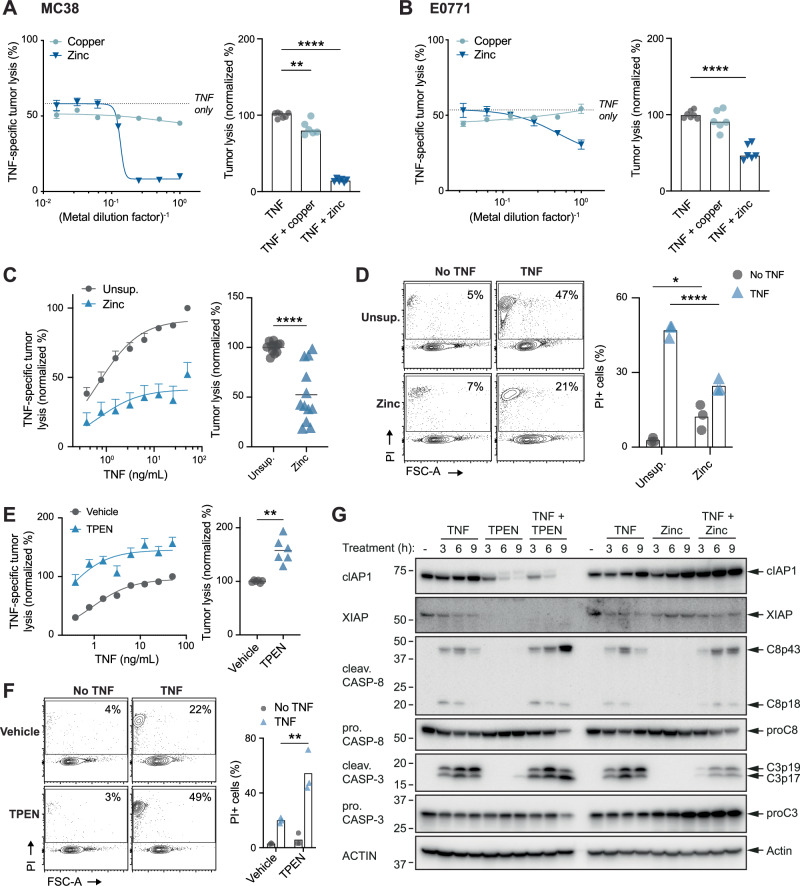
Zinc mediates tumour susceptibility to TNF-mediated cell death. Lysis of ^51^Cr-labelled MC38 tumour cells (**A**) and E0771 tumour cells (**B**) in 2-fold increasing concentrations of indicated metals in the presence of 10 ng/mL TNF over 16 h, using only non-toxic metal concentration determined in Supplementary Fig. S2 (i.e., metal concentrations causing <10% tumour lysis), Ordinary one-way ANOVA, pooled data *n* = 2. **C** Lysis of ^51^Cr-labelled MC38 tumour cells in increasing concentrations of TNF over 16 h in standard media or media supplemented with 100 µM ZnSO_4_. Right plot shows normalized percent tumour lysis at 50 ng/mL TNF, Mann–Whitney test, pooled data *n* = 4. **D** MC38 tumour cell death measured by PI uptake following 16 h of treatment with 10 ng/mL TNF in standard media or media supplemented with 100 µM zinc sulfate, 2way ANOVA, *n* = 3. **E** Lysis of ^51^Cr-labelled MC38 tumour cells in increasing concentrations of TNF over 16 h in media treated with 7 µM TPEN or the corresponding vehicle. Right plot shows normalized percent tumour lysis at 50 ng/mL TNF, Mann–Whitney test, pooled data *n* = 2. **F** MC38 tumour cell death measured by PI uptake following 7 h of treatment with 10 ng/mL TNF and 7 uM TPEN or the corresponding vehicle, 2way ANOVA, *n* = 3. **G** Immunoblot analysis of MC38 tumour cells following treatment with 10 ng/mL TNF with/without 7 µM TPEN or 70 µM ZnSO_4_ for the indicated time. All error bars show +/−SEM, *P < 0.05, **P < 0.01, ****P < 0.0001.

As CBFβ-deficient cells are resistant to TNF and exhibit transcriptional and post-transcriptional alterations in the homoeostasis of copper and zinc ions, we next investigated whether exogenous supplementation of these ions would alter tumour cell sensitivity to TNF. To determine the concentrations of copper and zinc that would lead to increased intracellular ion levels without inducing metal toxicity, we exposed both MC38 and E0771 cells to increasing concentrations of each ion (Supplementary Fig. S2A), and then selected concentrations at which tumour cell death was less than 10%, for use in subsequent assays. Next, to determine the effect of copper and zinc ion supplementation on tumour cell sensitivity to TNF, we treated MC38 and E0771 cells with TNF alone, or with serially diluted concentrations of each metal. While copper supplementation led to a small, albeit statistically significant, decrease in TNF sensitivity of MC38 cells across all doses, zinc supplementation led to a significant and substantial (>50%) dose-dependent decrease in the TNF sensitivity of both MC38 and E0771 cells (Fig. 3A, B). To further examine the role of zinc in modulating tumour cell sensitivity to TNF, we treated MC38 or E0771 cells with TNF, with or without zinc supplementation, and measured cell death by both chromium-release assays and PI uptake by flow cytometry. Consistent with our previous assays, the addition of zinc significantly decreased the sensitivity of both MC38 and E0771 to TNF-induced cell death, as measured by both TNF-specific lysis (i.e. normalized to any cell death occurring from zinc alone) (MC38; Fig. 3C, E0771; Supplementary Fig. S3A) and total cell death (Fig. 3D, Supplementary Fig. S3B). Together, these data confirmed that zinc can protect tumour cells from TNF-mediated cell death.

Given that zinc supplementation reduced tumour cell sensitivity to TNF, we next questioned whether sequestering zinc would contrariwise increase tumour cell TNF sensitivity. To test this, we analysed tumour cell death following treatment with TNF in combination with a membrane-permeable heavy metal chelator (tetrakis-(2-pyridylmethyl)ethylenediamine; TPEN) that has a high affinity for zinc. While TPEN alone had little to no effect on tumour cell viability, the addition of TPEN significantly increased TNF-mediated tumour cell death, measured as both TNF-specific lysis and total cell death in both MC38 and E0771 cells (MC38; Fig. 3E, F, E0771; Supplementary Fig. S3C, D).

Zinc is a well-known metal cofactor for inhibitor of apoptosis proteins (IAPs) [11], and it is well established that degradation or inhibition of IAPs sensitises cells to TNF induced apoptosis [12, 13]. We therefore questioned whether zinc chelation and zinc supplementation modulated tumour cell susceptibility to TNF through promoting the degradation or stabilisation of IAPs, respectively. To explore this, we measured IAP levels and caspase-3 and caspase-8 activation following treatment with TNF in combination with either TPEN or zinc supplementation. Strikingly, both cIAP1 and XIAP levels decreased considerably in as little as 3 h of treatment with TPEN (MC38; Fig. 3G, E0771; Supplementary Figs. S3E and S4). Consistent with this, the addition of TPEN also increased TNF-induced cleavage of caspase-3 and caspase-8 (Fig. 3G, Supplementary Figs. S3E and S4). In contrast, supplementation with zinc increased levels of cIAP1, prevented TNF-induced degradation of XIAP, and reduced TNF-induced cleavage of caspase-3 (Fig. 3G, Supplementary Figs. S3E and S4). Baseline levels of cIAP and XIAP were similar in MC38 sgCbfb cells and sgNT control cells, however, upon TNF treatment, neither cIAP or XIAP were degraded in sgCbfb MC38 cells. We also observed lower levels of both cleaved caspase-8 and -3 (and concomitant increase in pro-caspase-3 and -8) in *Cbfb*^*−/−*^ MC38 cells upon TNF treatment (Supplementary Fig. S5A). Furthermore, we found that TPEN fully restores TNF-mediated cytotoxicity in the absence of CBFβ (Supplementary Fig. S5B). Together, these data suggest that manipulation of intracellular zinc levels, through either supplementation or chelation, alters cellular levels of IAPs, which may in turn, modulate tumour cell susceptibility to TNF.

### Zinc protects tumours from T cell-mediated cytotoxicity

**Fig. 4:**
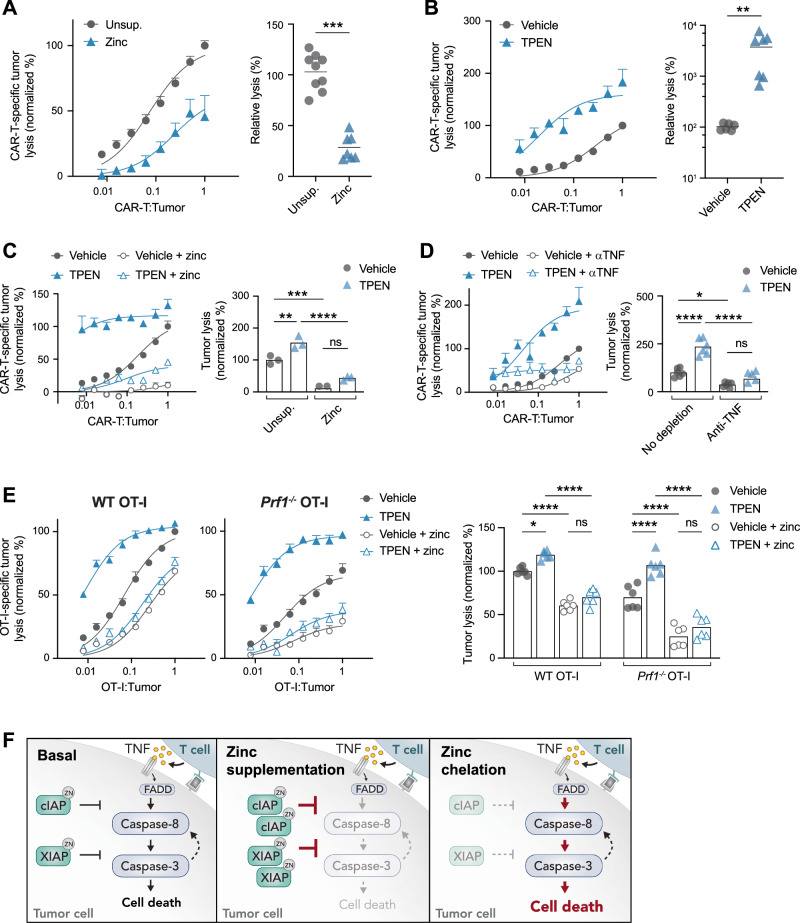
Zinc protects tumours from T cell-mediated cytotoxicity. **A**–**D** Lysis of ^51^Cr-labelled MC38-hHER2 tumour cells by hHER2-directed CAR-T cells in 16 h co-cultures, measured by ^51^Cr release at increasing CAR-T to tumour cell ratios. Co-cultures were treated with 7 µM TPEN or the corresponding vehicle in standard media or media supplemented with 100 µM ZnSO_4_ or TNF depletion antibodies. **A**, **B** Relative lysis is calculated as the efficiency of CAR-T cells to achieve an equal percent lysis of tumour cells, Mann–Whitney test, pooled data *n* = 3. **C**, **D** Right plots show normalized percent tumour lysis at a CAR-T to tumour cell ratio of 0.5:1, 2way ANOVA, pooled data *n* = 2. **E** Lysis of ^51^Cr-labelled MC38-OVA tumour cells by WT or perforin-deficient (*Prf*^*−/−*^) OT-I T cells in 16 h co-cultures, measured by ^51^Cr release at increasing OT-I to tumour cell ratios. Co-cultures were treated with 7 µM TPEN or the corresponding vehicle in standard media or media supplemented with 70 µM ZnSO_4_. **F** Proposed model of zinc modulation of tumour cell sensitivity to T cell-mediated cytotoxicity. All error bars show +/−SEM, *P < 0.05, **P < 0.01, ***P < 0.001, ****P < 0.0001.

Given the role of zinc in modulating tumour cell sensitivity to TNF, we next examined the effects of zinc modulation on tumour cell susceptibility to T cell killing. To do this, we co-cultured MC38-HER2 cells or E0771-HER2 cells with increasing numbers of HER2-directed CAR-T cells, with or without zinc supplementation or TPEN treatment. Consistent with our recombinant TNF cell death assays, zinc supplementation significantly decreased CAR-T cell specific lysis of tumour cells, while treatment with TPEN significantly increased tumour cell lysis (Fig. 4A, B, Supplementary Fig. S6A, B). To confirm that TPEN enhanced tumour susceptibility to CAR-T killing via zinc chelation, we re-supplemented TPEN-treated co-cultures with zinc and observed complete protection of tumour cells from the effects of TPEN (Fig. 4C, Supplementary Fig. S6C). Likewise, to confirm that TPEN enhanced CAR-T cell killing via increasing tumour cell susceptibility to TNF, we depleted TNF from co-cultures by the addition of a TNF-neutralising antibody. Indeed, TNF neutralisation completely abrogated the effects of TPEN on tumour cell sensitivity to CAR-T cell killing (Fig. 4D, Supplementary Fig. S6D).

Finally, we examined whether zinc modulation altered tumour cell susceptibility to direct perforin-mediated T cell killing. To do this, we co-cultured MC38-OVA cells with wildtype or perforin-deficient OT-I T cells, in combination with either zinc supplementation or TPEN treatment. Consistent with results observed in our CAR-T cell cultures, TPEN significantly enhanced T cell-specific tumour cell lysis, while zinc supplementation protected tumours from T cell killing and completely abrogated the effects of TPEN (Fig. 4E). Importantly, these results were the same in wildtype and perforin deficient-OT-I T cell cultures, demonstrating that the effects of zinc modulation on tumour cell sensitivity to T cell killing is independent of perforin. Together, these data support a model whereby intracellular zinc modulation, via supplementation or chelation, modulates tumour cell sensitivity to T-cell-derived TNF (Fig. 4F).

## Discussion

In this study we aimed to uncover novel regulators of tumour immune-evasion by identifying genes that promote tumour cell susceptibility to MHC-unrestricted CAR-T cell cytotoxicity. In line with previous studies by us and others, we identified TNF-mediated cell death as a critical mechanism by which cytotoxic T cells elicit their anti-tumour activity [5–7, 14]. We further identified the transcription factor, CBFβ, as a positive regulator of tumour sensitivity to TNF-mediated cell death. CBFβ has a well-established role in haematopoiesis and leukaemogenesis [15–17]. While its role in solid cancers is less defined, existing studies implicate CBFβ in the promotion of solid cancer growth, plasticity, and metastasis [8, 18, 19]. To our knowledge, our study has uncovered the first mechanism by which CBFβ attenuates tumour cell fitness, by enhancing vulnerability to immune clearance. Importantly, previous studies evaluating the role of CFBβ in solid tumours have been performed in human model systems in the absence of an immune system, likely explaining why a role for CBFβ in immune evasion has not been identified previously. Our findings showed that CBFβ depletion lowered the tumour TNF toxicity threshold, rather than completely abrogating TNF sensitivity. This suggests that CBFβ is not a critical component of the TNF-induced death pathway, but more likely modulates TNF sensitivity indirectly. To determine why CBFβ-depleted cells were less susceptible to TNF, we performed RNA-seq across two mouse cancer cell lines and compared our results to available published data in CBFβ-deficient human tumour cells [8]. We identified that alteration of metal ion homoeostasis is a common feature of tumour cells lacking CBFβ although the underlying molecular basis remains to be defined. Further analyses showed that modulation of cellular zinc dramatically impacted tumour susceptibility to TNF.

Zinc and other metals play an important role in cellular function by acting as essential cofactors in thousands of intracellular proteins, where they contribute to regulation of protein stability and/or function [20–22]. Accordingly, cellular metal homoeostasis is tightly regulated by complex metalloregulatory networks that control the expression of genes that include membrane transporter and intracellular metal-binding proteins, such as Mt. Disrupted metal ion homoeostasis is commonly observed in cancer, and dysregulated zinc, copper, and iron have been shown to promote cancer initiation, survival, progression, and metastasis [23–26]. While less is known about the role of metal homoeostasis in tumour immune evasion, copper has been shown to protect tumours from T cell killing via upregulating expression of the T cell inhibitory ligand, PD-L1 [27]. Indeed, treatment of mice with copper-chelating agents, dextran-catechin (DC) or tetraethylenepentamine TEPA, increased cytotoxic lymphocyte infiltration into tumours and slowed tumour growth, suggesting that targeting tumour ion homoeostasis has potential as an anti-cancer immunotherapeutic. Here, we observed intracellular increases in the levels of metals that play crucial roles in regulating cellular activity (magnesium and calcium), contribute to oxidative stress management (manganese, copper, zinc, and selenium) [28], regulate kinase activity and enable mitochondrial respiration (copper) [29], and metalloprotein structure and/or activity (zinc) [30]. In cells lacking CBFβ, intracellular increases in these metals occurred concomitantly with a downregulation in the expression of Mt genes. Reducing the abundance of this crucial buffer for intracellular copper and zinc would increase the labile pool of these metals and thereby promoting their ability to interact with permissive proteins, such as IAPs. Our findings are consistent with the zinc serving as a stabilising cofactor for these proteins, as survival was enhanced by further supplementing the cellular pool of the metal [11]. Further, decreasing cellular zinc via chelation increased tumour cell susceptibility to TNF-mediated death in a similar manner to therapeutic IAP antagonists (also known as smac-mimetics).

Our observation that zinc chelation destabilises IAPs suggest that these proteins are particularly sensitive to changes in intracellular zinc levels. However, whether other proteins, including other zinc-finger proteins, are similarly sensitive to zinc chelation requires further investigation. Indeed, the sensitivity of specific proteins to intracellular zinc may depend on their specific structural characteristics and function. Evaluating the effects of zinc chelation on other proteins and its broader impact on cellular processes is essential to ensure the development of safe and effective therapeutic strategies. Promisingly however, while smac-mimetics primarily target cIAPs, the dual action of zinc chelation in degrading both cIAPs and XIAPs may make it a more potent and comprehensive approach to sensitise tumour cells to TNF-induced apoptosis. Hence, as a therapeutic strategy, zinc chelation could potentially offer a promising advantage over smac-mimetics.

Taken together, our results demonstrate that intracellular zinc protects tumour cells from TNF-mediated cell death through the stabilisation of IAPs. This zinc dysregulation renders tumours less sensitive to immune clearance by T cells by increasing the toxicity threshold of T cell-derived TNF. Zinc homoeostasis may therefore present a novel tumour cell vulnerability that may be targeted therapeutically for the treatment of cancer.

## Materials and methods

### Mice

C57BL/6 were purchased from Animal Resource Centre (WA) and C57BL/6 OT-I transgenic mice were bred in house. All mice were housed in the Peter MacCallum Cancer Centre Animal Core Facility under specific pathogen-free conditions.

### Antibodies and reagents

Anti-CBFβ (Ab33516) was purchased from Abcam (Cambridge, United Kingdom). Secondary antibody was polyclonal swine anti-rabbit, purchased from DAKO (Jena, Germany), Propidium Iodine (PI) (Sigma-Aldrich, Sydney, New South Wales, Australia). Recombinant mouse TNF was purchased from PeproTech (Rocky Hill, New Jersey, USA).

### Cell lines

Murine MC38 and E0771 cell lines were cultured in DMEM (Gibco, Melbourne, Victoria, Australia; Invitrogen, Scoresby, Victoria, Australia) supplemented with 10% (v/v) fetal calf serum (FCS) (Thermo Scientific, Scoresby, Victoria, Australia) and penicillin/streptomycin (Gibco) and incubated at 37 °C, 10% CO_2_. All lines were verified to be mycoplasma-negative by the Victorian Infectious Diseases Reference Laboratory by PCR analysis. For generation of edited tumour cell lines, the cells were transiently transfected using the Amaxa Nucleofector 4D System (Lonza) using a synthetic guide (IDT) and Cas9 Nuclease V3 (IDT). Oligonucleotides used: Cbfb GCCTTGCAGATTAAGTACAC; Negative control (non-targeting) GCACUACCAGAGCUAACUCA.

### Mouse T cells

The generation of murine CAR-T cells was done as previously described [31, 32]. Briefly, retrovirus encoding a CAR composed of an extracellular scFv–anti–human HER2 fused to the transmembrane domains of CD28 and CD3ζ was transduced into anti-CD3 and anti-CD28 activated T cells from the spleen of C57BL/6 mice. Primary OT-I T cells were isolated from the spleens of C57BL/6 OT-I transgenic mice and activated with OVA_257_ peptide (Auspep, Tullamarine, Victoria, Australia). All T cells were cultured in enriched T cell media (RPMI supplemented with 10% (v/v) FCS, penicillin/streptomycin, L-glutamine, non-essential amino acids, 4-(2-hydroxyethly)-1-piperazineethanesulfonic acid (HEPES), sodium pyruvate (Calbiochem, Macquarie Park, New South Wales, Australia) and 100 IU/mL IL-2 (ROCHE, New South Wales, Australia) and 2 ng/mL IL-7 (CAR-T cells only) (PeproTech, Rocky Hill, NJ). Cells were incubated at 37°C, 5% CO_2_ and routinely used on day 4–8 post-activation.

### Cytotoxicity assays

The cytotoxic activity of T cells was measured using a standard chromium release assay as previously described [33]. The percentage specific killing was determined using the formula: (Sample ^51^Cr release – Spontaneous ^51^Cr release)/ (Total ^51^Cr release − Spontaneous ^51^Cr release) × 100. All assays were performed using triplicate wells. To generate relative killing (fold) graphs, relative killing at the E:T ratio that resulted in 50% maximal killing of the least cytotoxic condition was compared using Michaelis-Menten trends, as described previously [33].

### Competition assays

Tumour cells were transduced with lentivirus containing lentiGuide-Crimson (Addgene Plasmid: 70683) in the presence of sequa-brene (5 μg/mL; Sigma-Aldrich). Virus was generated from overnight transfection of HEK293T cells in the presence of polyethylenimine using 3^rd^ generation lentiviral envelope and packaging plasmids: pMD2.G, pMDL, and pRSV-Rev. 48 h after transfection, virus was filtered and then added to adhered tumour cells targets. Forty-eight hours after transduction, crimson^+^ cells were FACS sorted in-house at the Peter MacCallum Flow Cytometry Core Facility using a FACS Aria Fusion flow cytometer (BD Biosciences).

### Sequencing

3′ mRNA sequencing: Tumour cells were lysed and total RNA was isolated as per the manufacturer’s instructions (NucleoSpin RNA Extraction kit, Macherey-Nagel, Bethlehem, PA). Subsequently, the RNA was analysed on a TapeStation (Agilent TapeStation 2200), and only RNA with RNA integrity number values greater than 9 were used for downstream library preparation. The QuantSeq 3′ mRNA Library Prep Kit (Lexogen) was used to prepare libraries. The 3′ mRNA sequencing libraries were sequenced single-end 75 base pairs (bp) on the NextSeq 500 (Illumina). The resulting reads were demultiplexed using CASAVAv1.8.2, and sample quality control was performed using FastQC (Babraham Bioinformatics, Babraham Institute). The reads were trimmed using cutadapt (v1.9) and subsequently aligned to the mouse reference genome (GRCm38/mm10) using HISAT2 (v2.1.0), after which read counting was performed using FeatureCounts from the Subread package (v1.5.0). Differential gene expression analysis was performed using Voom-LIMMA.

### CRISPR screen

Tumour cells were transduced with a mouse CRISPR Knockout Pooled Library (Brie) (Addgene Cat: 73633). Transductions were performed at a multiplicity of infection of 0.3 to ensure integration of single sgRNA constructs per cell. After transduction, transduced cells were selected with puromycin (6 μg/mL) for 5 days and then co-cultured with HER2-targeting mouse CAR-T cells at effector to target (E:T) ratios of 2:1. The tumour cells were exposed to a total of three rounds of CAR-T cell killing, after which the pellets were snap-frozen. Genomic DNA extraction was subsequently performed using the DNeasy Blood & Tissue Kit (Qiagen), and libraries were prepared. The libraries were subsequently multiplexed and run on the NextSeq 500 (Illumina) generating 75-bp single-end reads. After demultiplexing with CASAVA (v1.8), the vector-derived sequence reads were removed, and only reads of exactly 20 bp were extracted using cutadapt (v1.7). Subsequently, MAGeCK (v0.5.6) was used to count the reads and perform gene/sgRNA enrichment and statistical analysis. The resulting data were visualized using the R package ggplots2.

### Western blotting

To analyse proteins, we used NaDodSO4 PAGE (SDS-PAGE). Samples were lysed in radioimmunoprecipitation assay (RIPA) buffer on ice for 30 min, and supernatants were collected following 10 min of centrifugation at 10,000 × *g*. Lysates were then loaded into 8–12% polyacrylamide gels and electrophoresed at 100 V. Resolved proteins were then transferred onto nitrocellulose membranes at 40 mA overnight. Membranes were blocked for 1 h (5% (w/v) BSA, 0.05% (w/v) NaN_3_ in PBS, Tween 20), then incubated with primary antibody overnight. Proteins were detected using HRP-conjugated secondary antibody, using the ECL system (Amersham Bioscience) according to the manufacturer’s protocols using an iBright FL1500 imaging system (Thermo Fisher Scientific).

### ICP-MS

Tumour cells were trypsinized and counted, and equal numbers of cells were collected per sample. Samples were washed 3 times with PBS by centrifugation for 4 min at 400 × *g* and cell pellets were stored at room temperature for ICP-MS. Cell pellets were digested by addition of 250 μL of 65% (v/v) HNO_3_ (Suprapur, Merck) followed by heating at 96 °C for 20 min. Samples were allowed to cool, briefly vortexed, centrifuged at 20,000 × *g* for 25 min and 50 μL of supernatant was diluted in MilliQ-H_2_O to a final volume of 1 mL. Samples were prepared in technical duplicate. Elemental analysis was performed using an Agilent 8900 triple quadrupole ICP-MS (Agilent Technologies, Mulgrave, Australia). The ICP-MS was calibrated using 0, 1, 2.5, 5, 10, 25, 50, 100, 250, 500 and 1000 parts per billion (ppb) of a certified multi-element calibration standard (Agilent Technologies). A certified standard solution containing 100 ppb of yttrium (Agilent Technologies), via T-piece introduction, was used as an internal standard. PBS and sample preparation blanks (containing MilliQ-H_2_O and HNO_3_) were measured to monitor for elemental contamination. Data expressed as molar concentration. Operating parameters for Agilent 8900 triple quadrupole ICP-MS were as follows:

**Table:** 

Instrument parameters	
Scan type	Single quad
Cell gas flow	He, 5.0 mL min^−1^
Nebulizer	MicroMist
Nebulizer gas flow	1.05 L min^−1^
RF power	1550 W
Sample depth	8.0 mm
Spray chamber temperature	2 °C
Extracts 1, 2	−12.0, −250.0 V
Omega bias, lens	−130, 6.0 V
Cell entrance, exit	−50, 60 V
Deflect, plate bias	−5.4, −60 V
OctP bias, RF	−18.0, 180 V
Q1 entrance, exit	−50.0, 0.0 V
Q1 bias	−3.0 V
Measured masses	24, 43, 44, 55, 56, 59, 60, 63, 66, 78, 89 & 111

## Supplementary information

Supplemental Material

## Data Availability

Sequencing data has been deposited the GEO repository under Accession Number GSE244141.
