# Supplementary material for: Intracellular zinc protects tumours from T cell-mediated cytotoxicity
Source: Cell Death Differ. 2024 Sep 11;31(12):1707–16. doi: 10.1038/s41418-024-01369-4 (PMC11618339; doi:10.1038/s41418-024-01369-4)

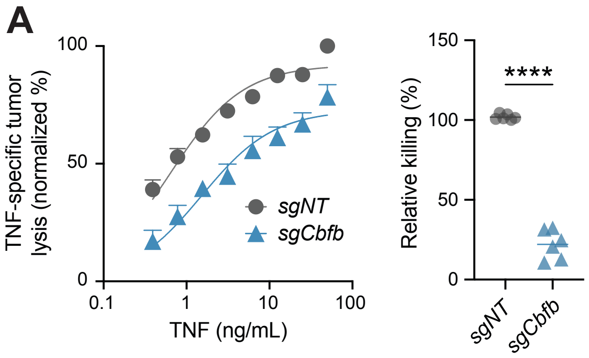

Figure S1. (A) Lysis of 51Cr-labelled E0771 tumor cells in increasing concentrations of TNF over 16 h. Relative lysis is calculated as the efficiency of TNF to achieve an equal percent lysis of tumor cells, unpaired t test, n=6. Error bars show +/- SEM, **** P <0.0001.

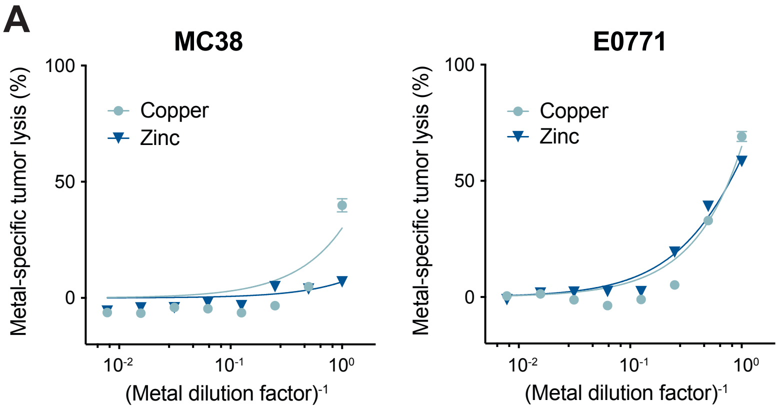

Figure S2. (A) Lysis of 51Cr-labelled MC38 and E0771 tumor cells in 2-fold increasing concentrations of indicated metals over 16 h, starting at top concentrations of 2 mM CuSO4 sulfate, 2 mg/mL ferric ammonium citrate (iron) and 400 µM ZnSO4, representative of n=2.

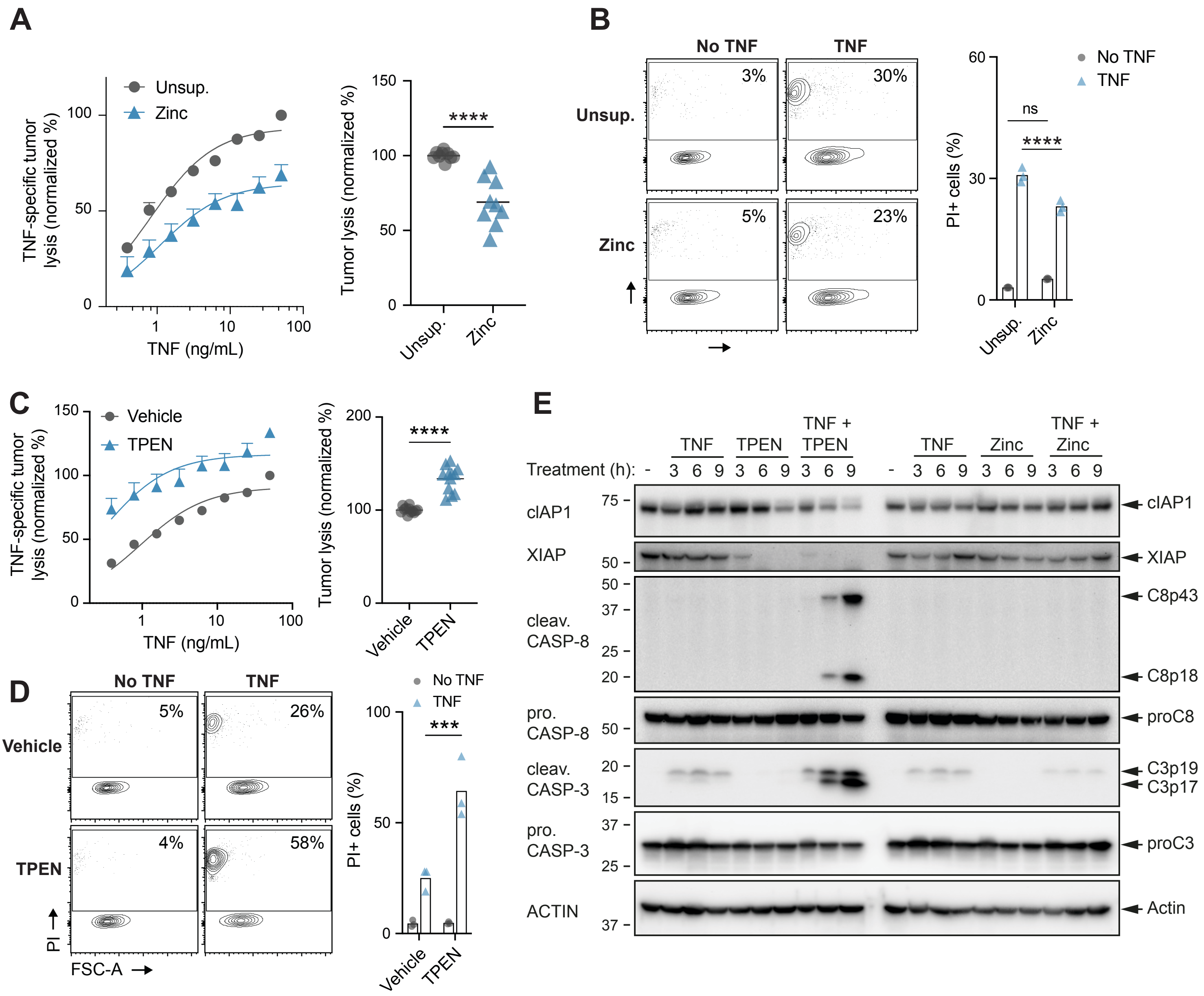

Figure S3. (A) Lysis of 51Cr-labelled E0771 tumor cells in increasing concentrations of TNF over 16 h in standard media or media supplemented with 70 µM ZnSO4. Right plot shows normalized percent tumor lysis at 50 ng/mL TNF, Mann-Whitney test, pooled data n=3. (B) E0771 tumor cell death measured by PI uptake following 16 h of treatment with 10 ng/mL TNF in standard media or media supplemented with 70 µM ZnSO4, 2way ANOVA, n=3. (C) Lysis of 51Cr-labelled E0771 tumor cells in increasing concentrations of TNF over 16 h in media treated with 7 µM TPEN or the corresponding vehicle. Right plot shows normalized percent tumor lysis at 50 ng/mL TNF, Mann-Whitney test, pooled data n=4. (D) E0771 tumor cell death measured by PI uptake following 7 h of treatment with 10ng/mL TNF with 7 µM TPEN or the corresponding vehicle, 2way ANOVA, n=3. (E) Immunoblot analysis of E0771 tumor cells following treatment with 10 ng/mL TNF with/without 7 µM TPEN or 70 µM ZnSO4for the indicated time. All error bars show +/- SEM, *** P< 0.001, **** P <0.0001.

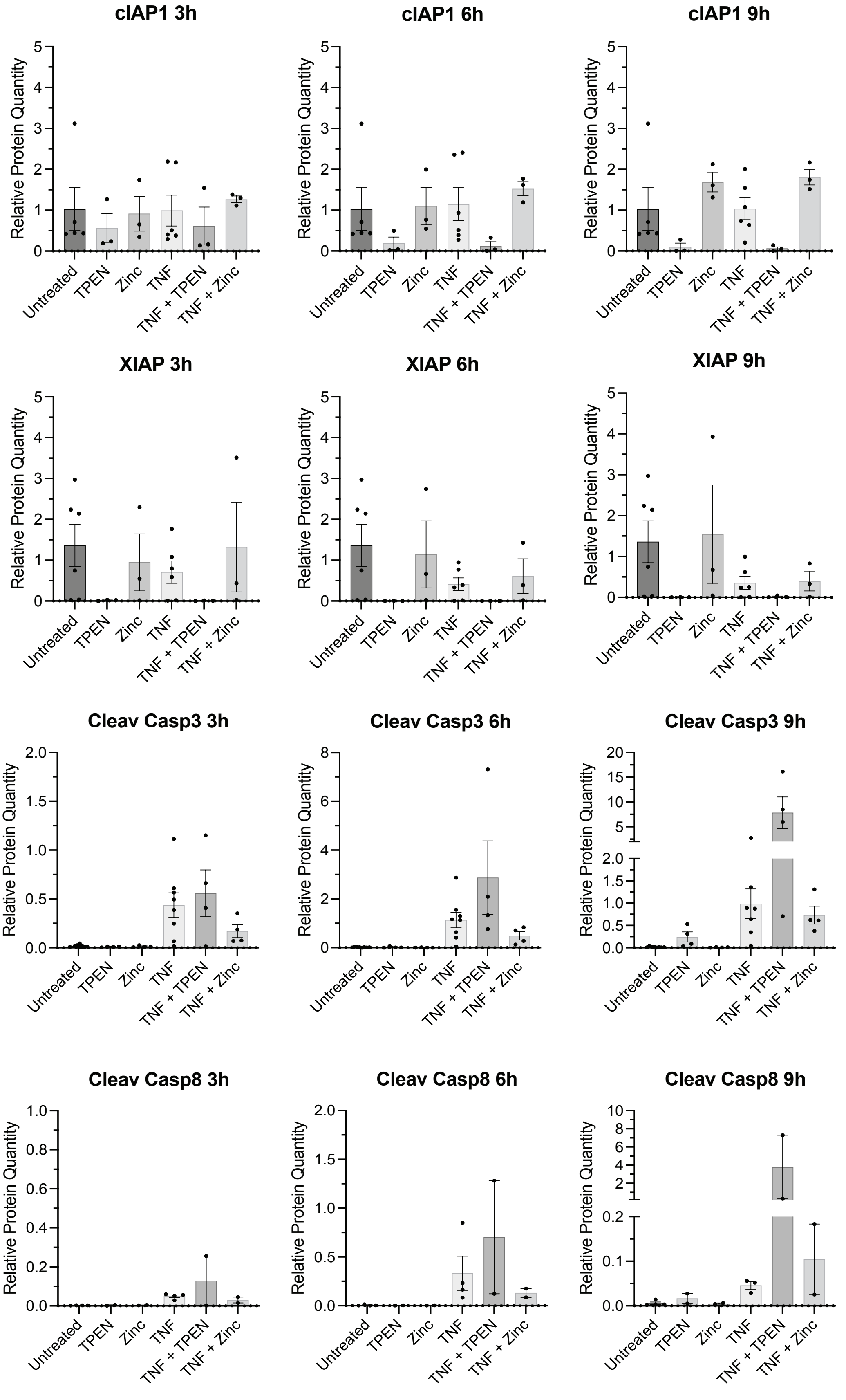

Figure S4. Quantification of protein from replicates of Western blot presented in Figure 3G (n=2-6 for each protein) for MC38 cells.

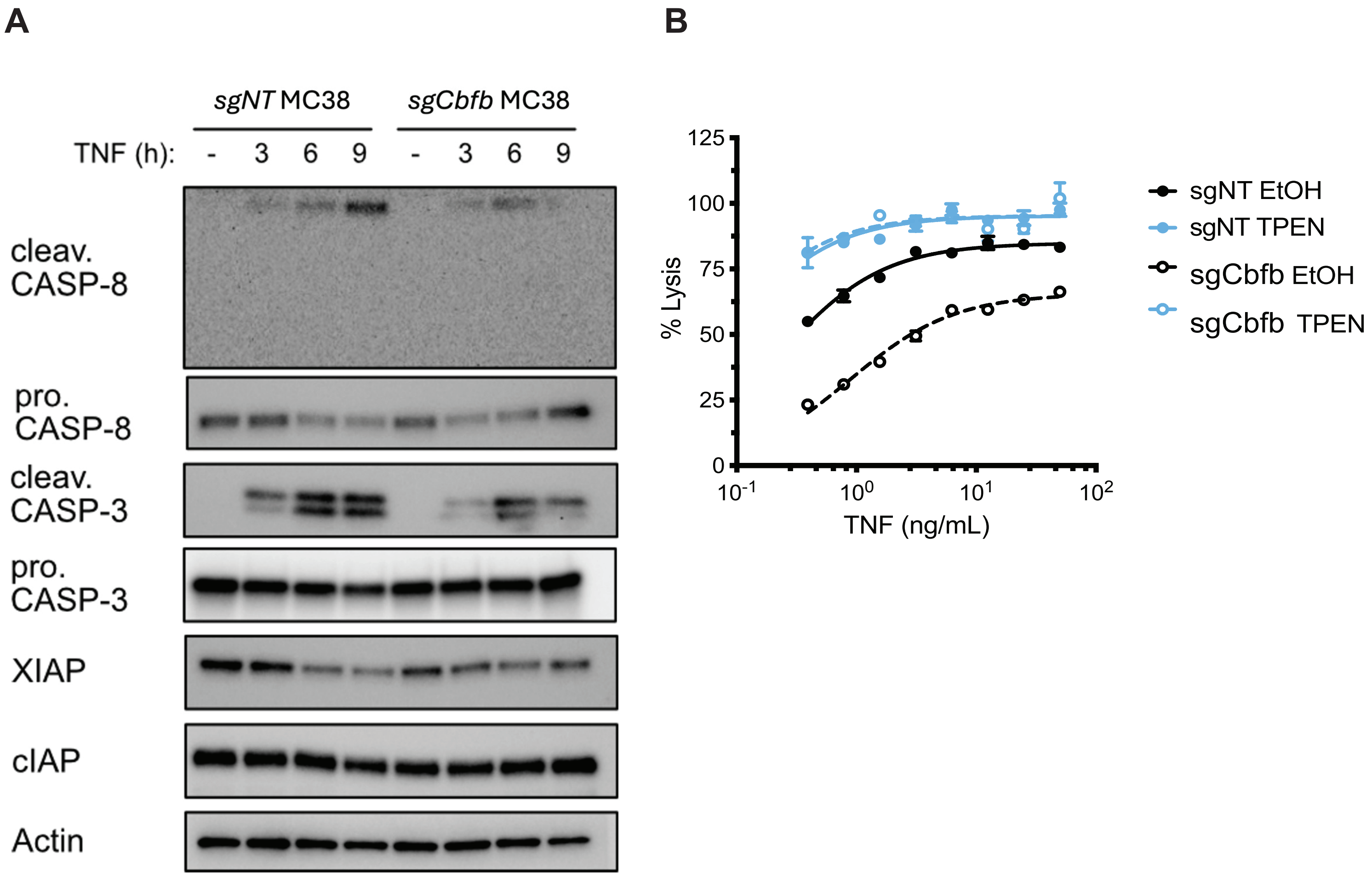

Figure S5. (A) Immunoblot analysis of indicated proteins in wildtype (sgNT) and CBFB deficient (sgCbfb) MC38 tumor cells following treatment with 10 ng/mL TNF for the indicated time. (B) Lysis of 51Cr-labelled wildtype (sgNT) and CBFB deficient (sgCbfb) MC38 tumor cells in increasing concentrations of TNF over 16 h in media treated with 7 μM TPEN or the corresponding vehicle (EtOH).

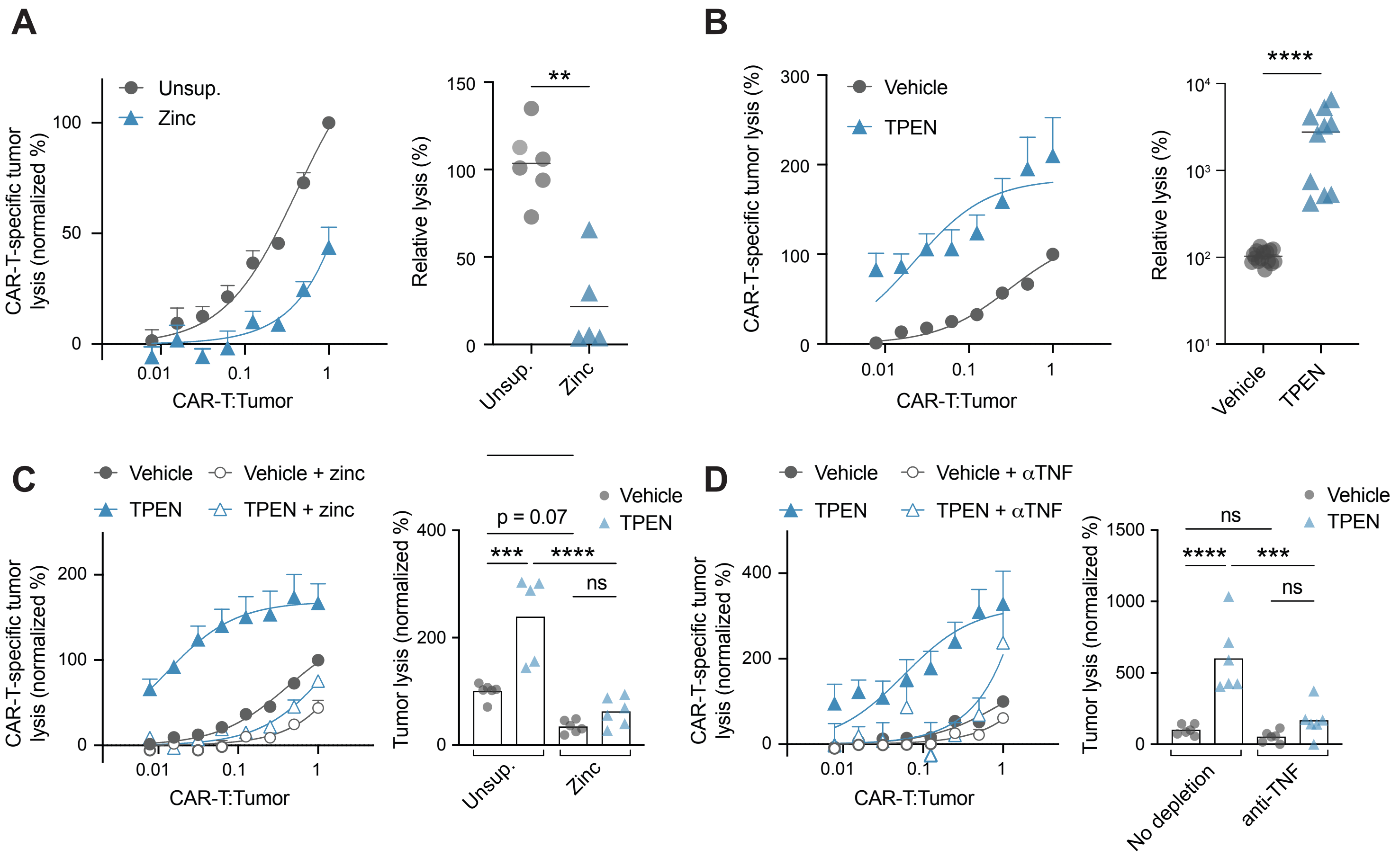

Figure S6. (A-D) Lysis of 51Cr-labelled E0771-hHER2 tumor cells by hHER2-directed CAR-T cells in 16 h co-cultures, measured by 51Cr release at increasing CAR-T to tumor cell ratios. Co-cultures were treated with 7 µM TPEN or the corresponding vehicle in standard media or media supplemented with 100 µM ZnSO4or TNF depletion antibodies. (A-B) Relative lysis is calculated as the efficiency of CAR-T cells to achieve an equal percent lysis of tumor cells, Mann-Whitney test, pooled data n=3. (C-D) Right plots show normalized percent tumor lysis at a CAR-T to tumor cell ratio of 0.5:1, 2way ANOVA, pooled data n=2. All error bars show +/- SEM, ** P< 0.01, *** P<0.001, **** P <0.0001.

Lelliiott et al. Original Westerns Figure 3

cIAP1

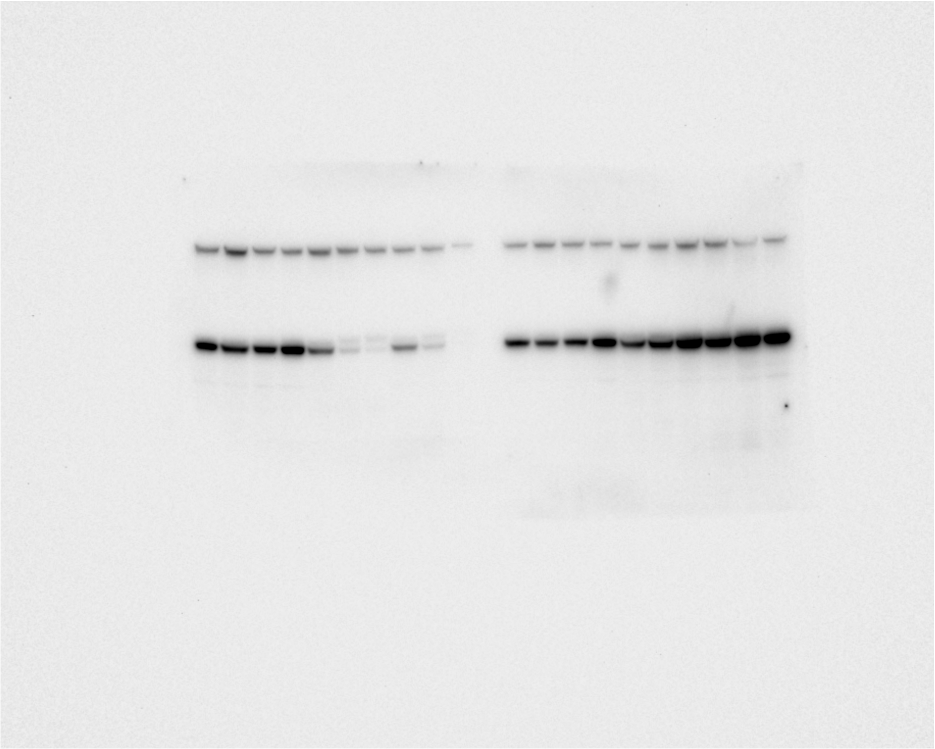

XIAP

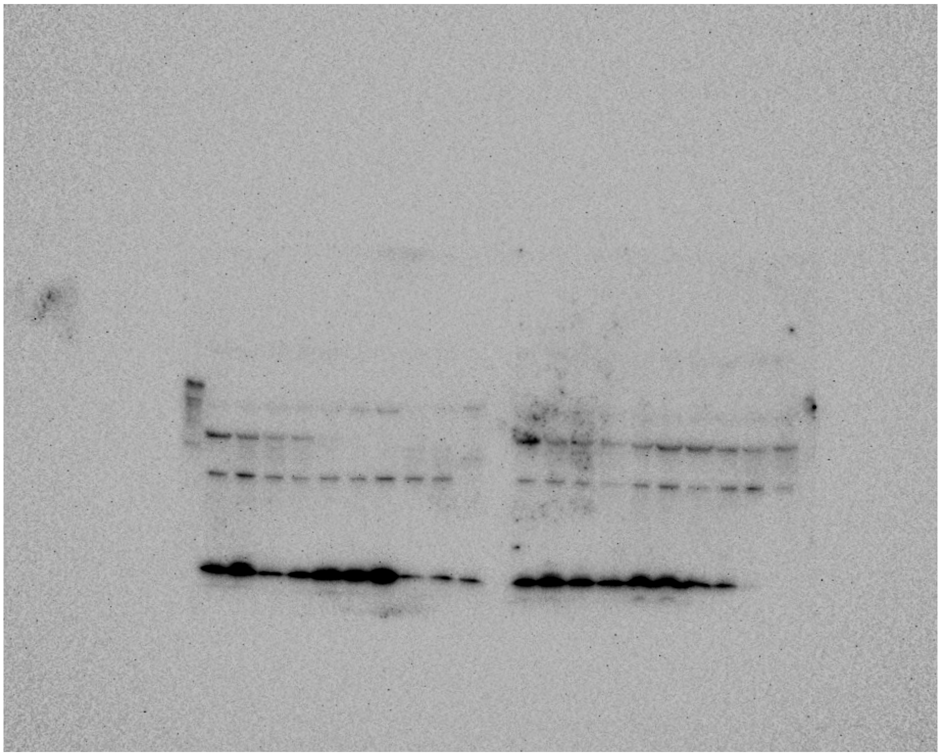

Lelliiott et al. Original Westerns Figure 3

Pro-Caspase 8

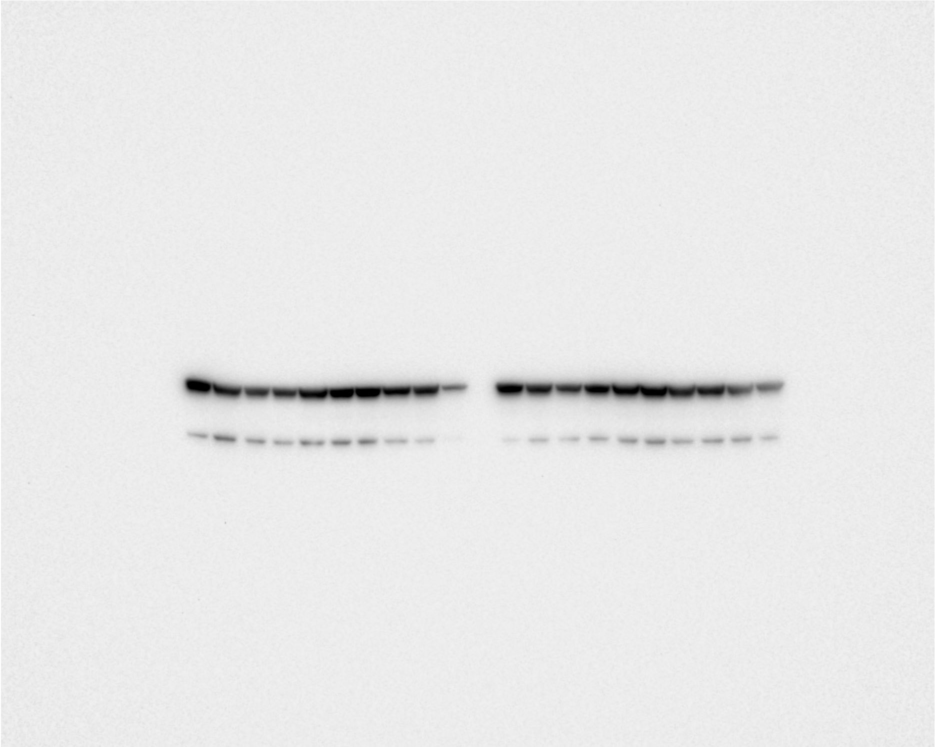

Cleaved Caspase 8

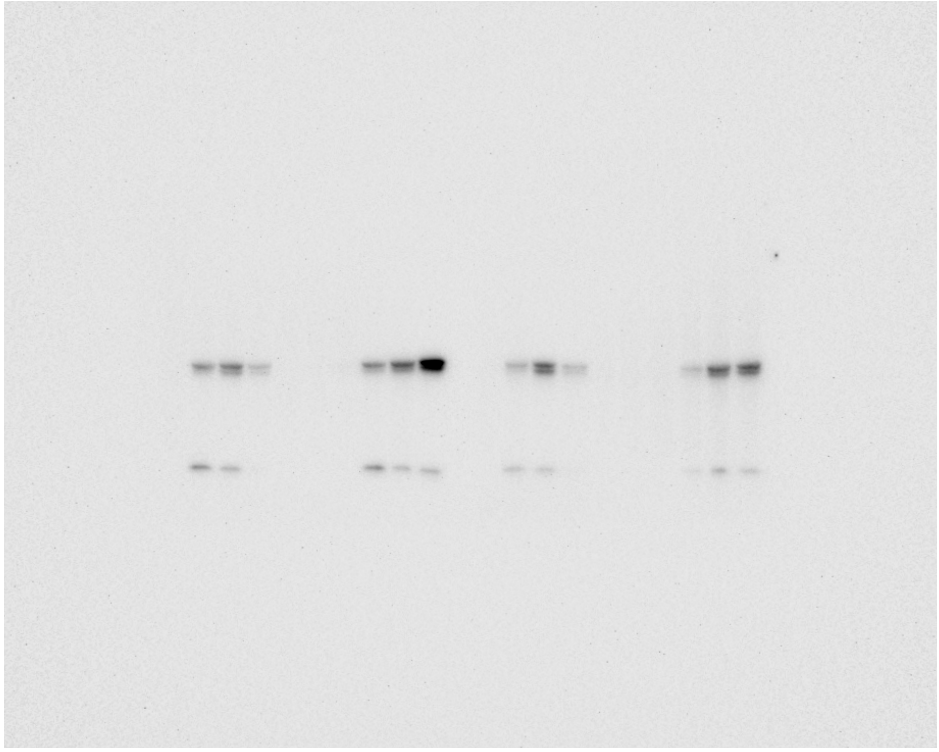

Lelliiott et al. Original Westerns Figure 3

Pro-Caspase 3

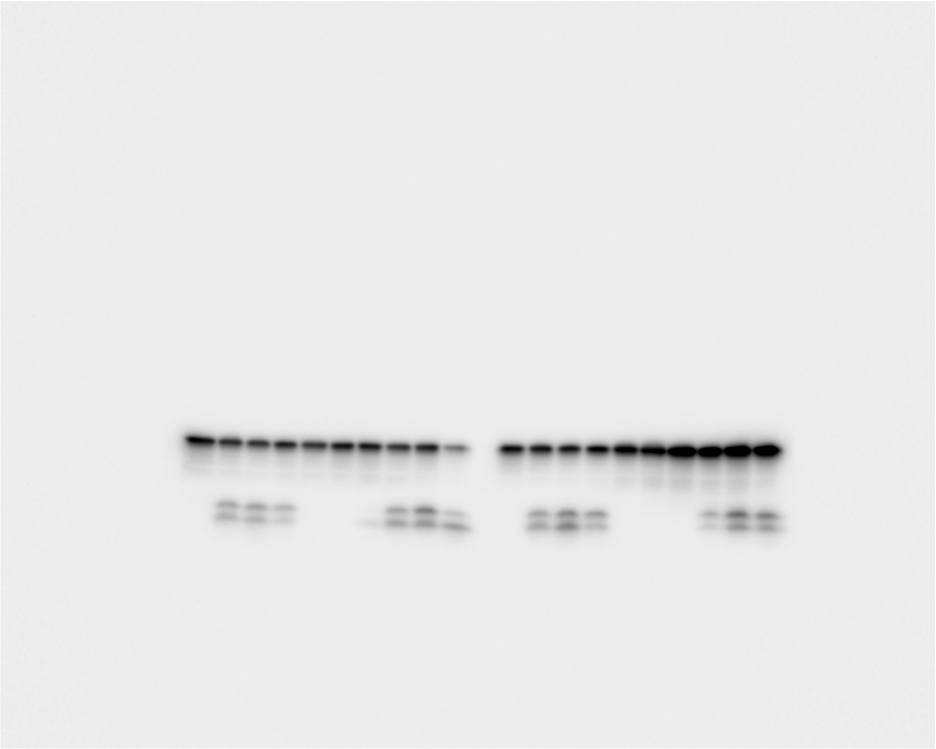

Cleaved Caspase 3

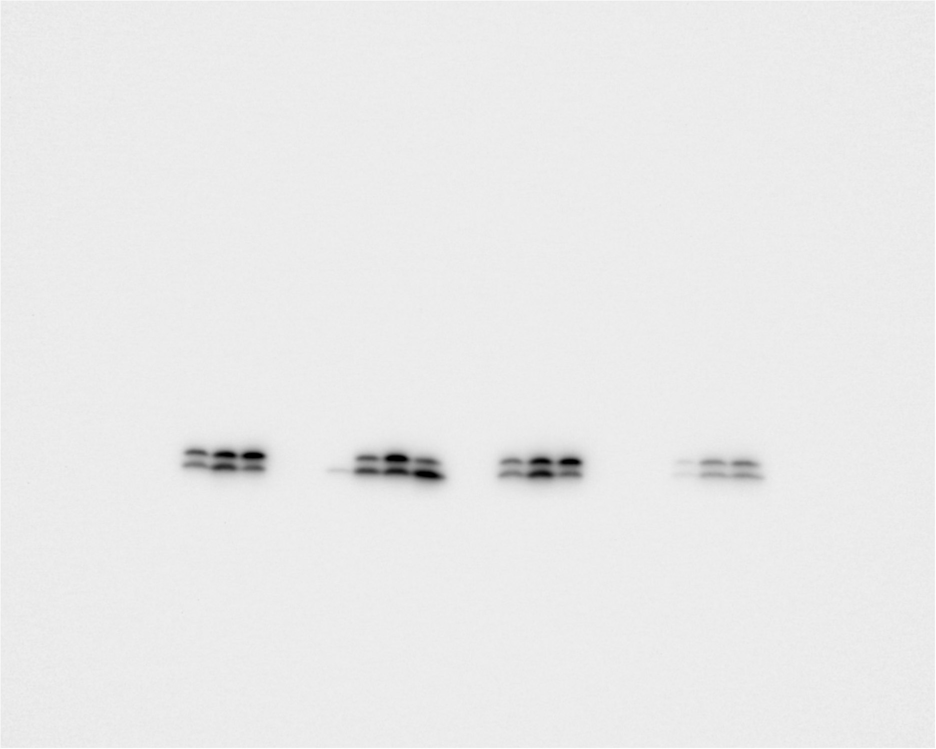

Actin

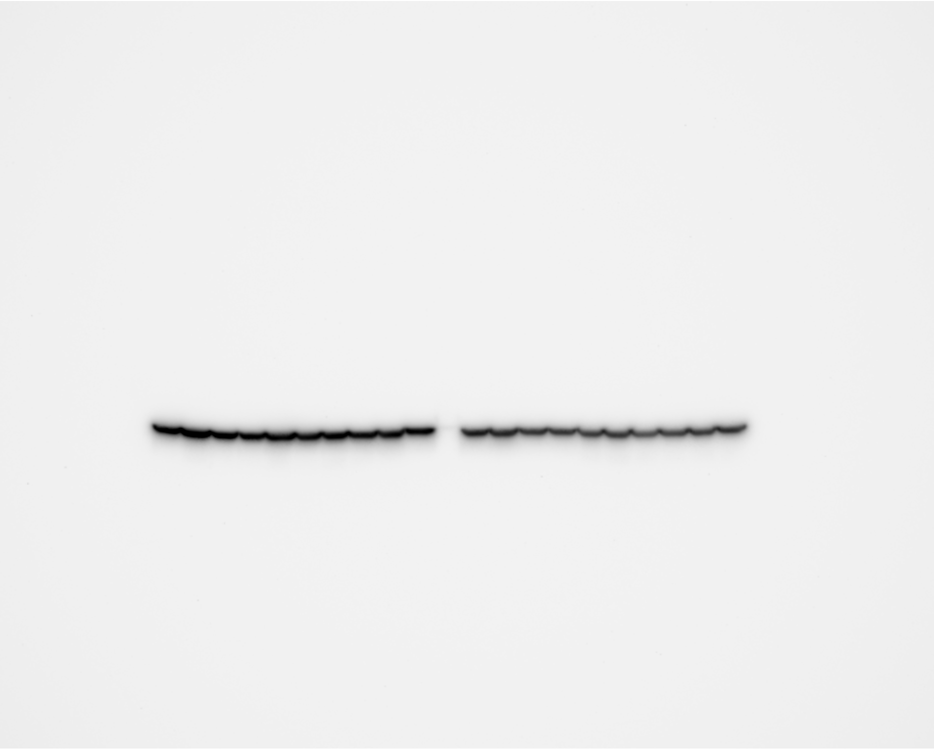

Lelliott et al. Original Westerns Supplementary Figure 3

cIAP1

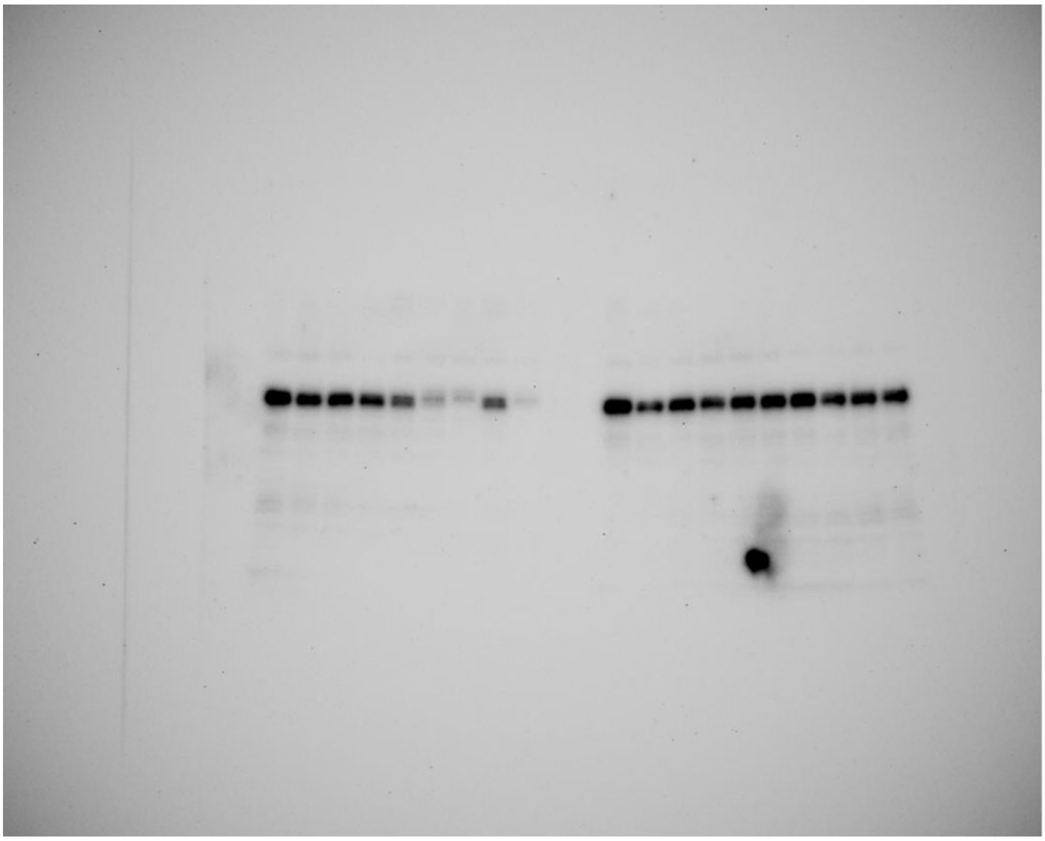

XIAP

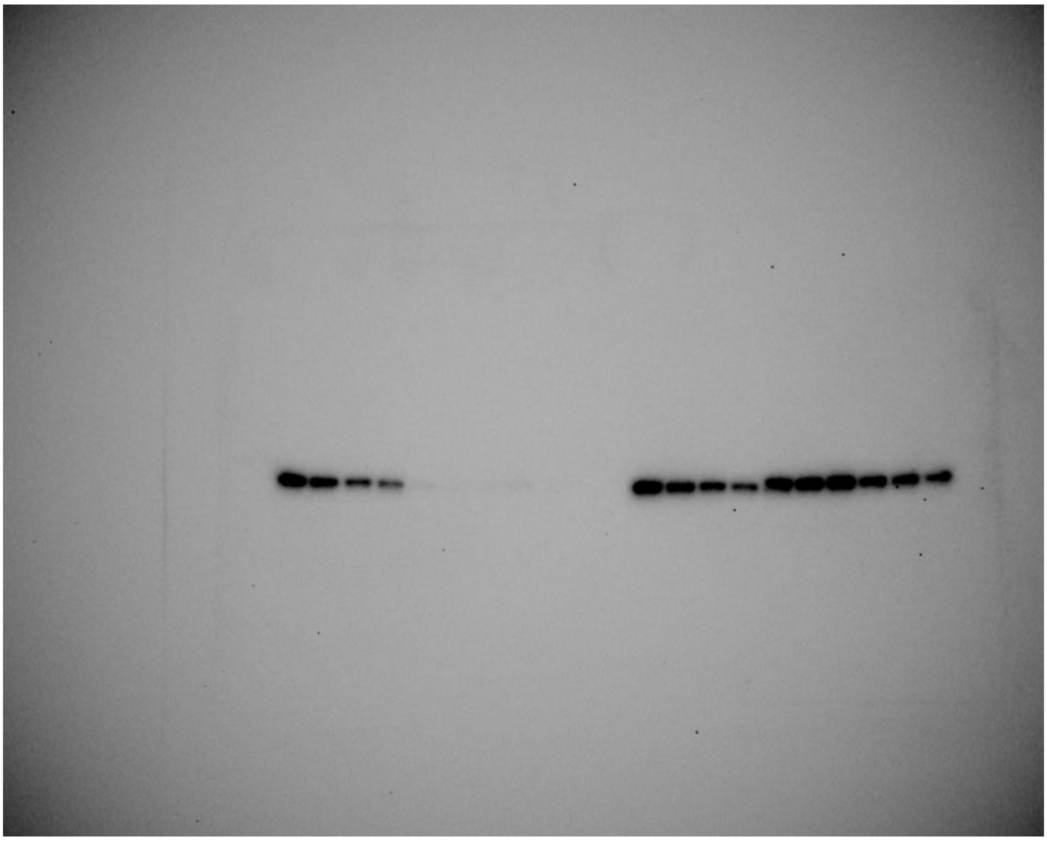

Lelliott et al. Original Westerns Supplementary Figure 3

Cleaved Caspase-3

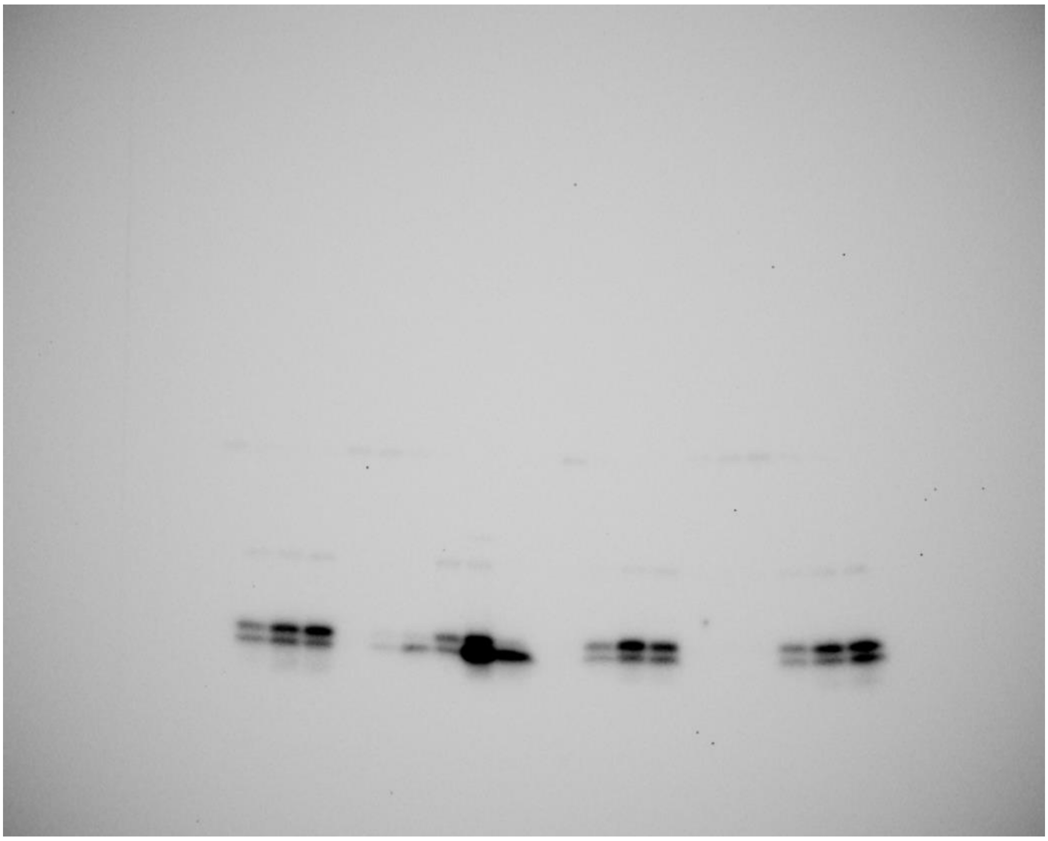

Actin

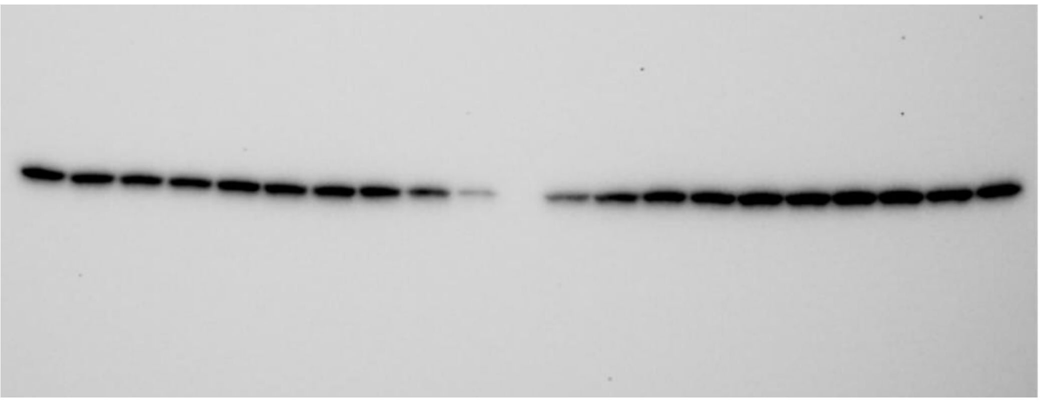

Lelliott et al. Original Westerns Supplementary Figure 3

cIAP1

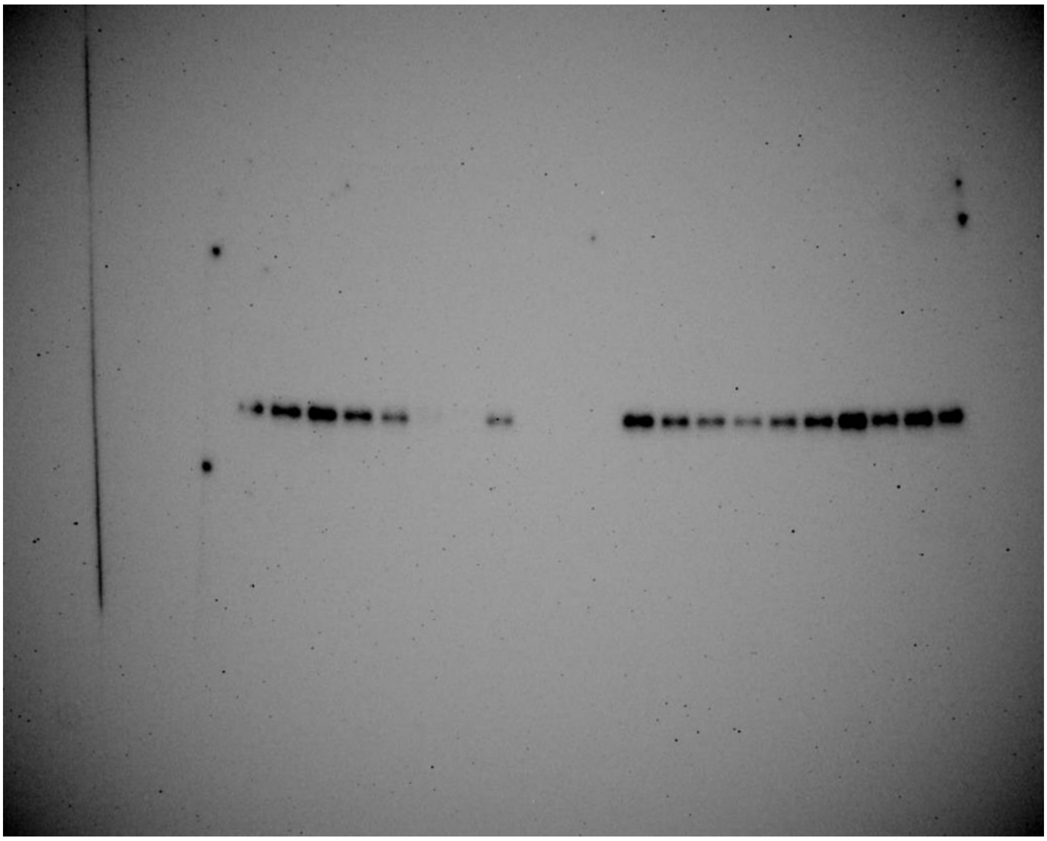

XIAP

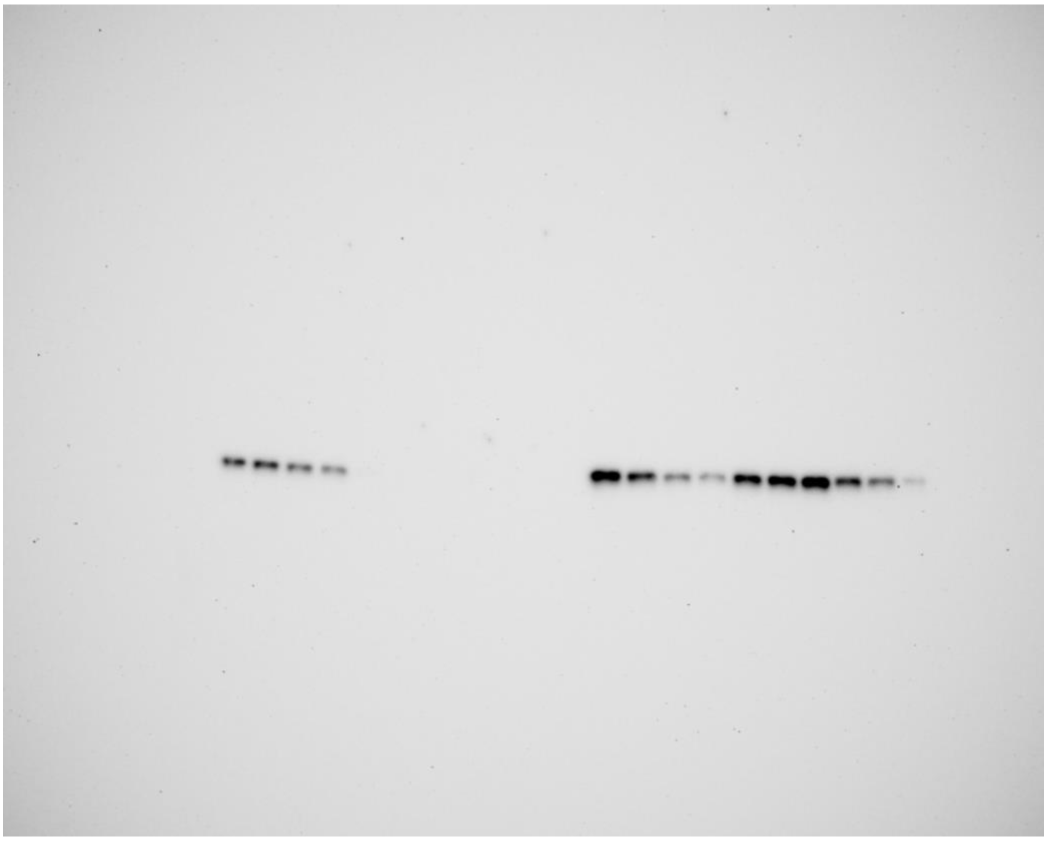

Lelliott et al. Original Westerns Supplementary Figure 3

Cleaved Caspase-3

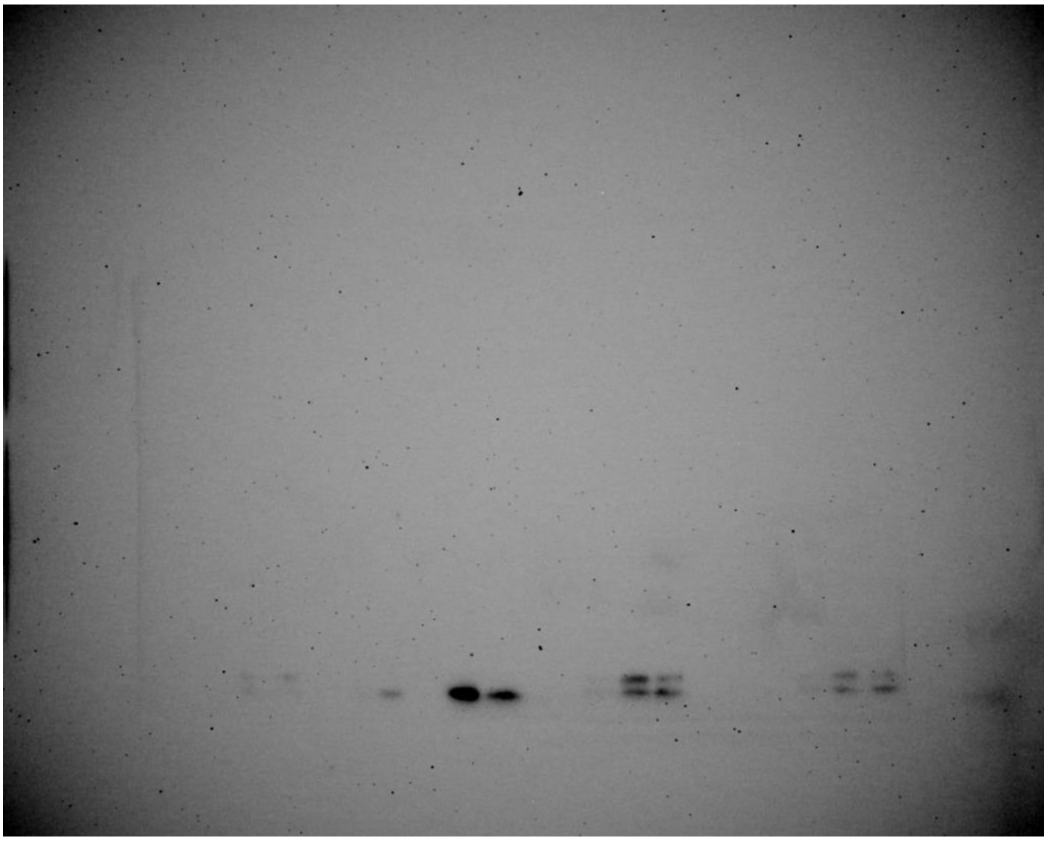

Actin

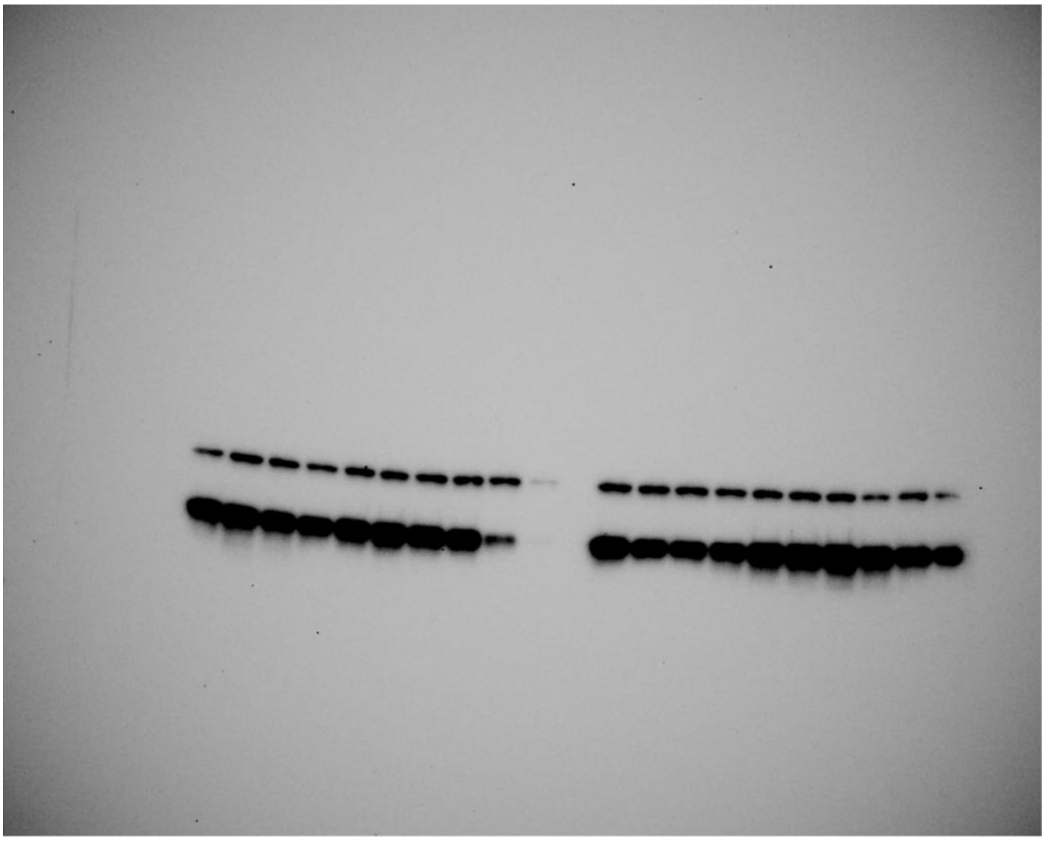

Lelliott et al. Original Westerns Supplementary Figure 3

Cleaved Caspase-8

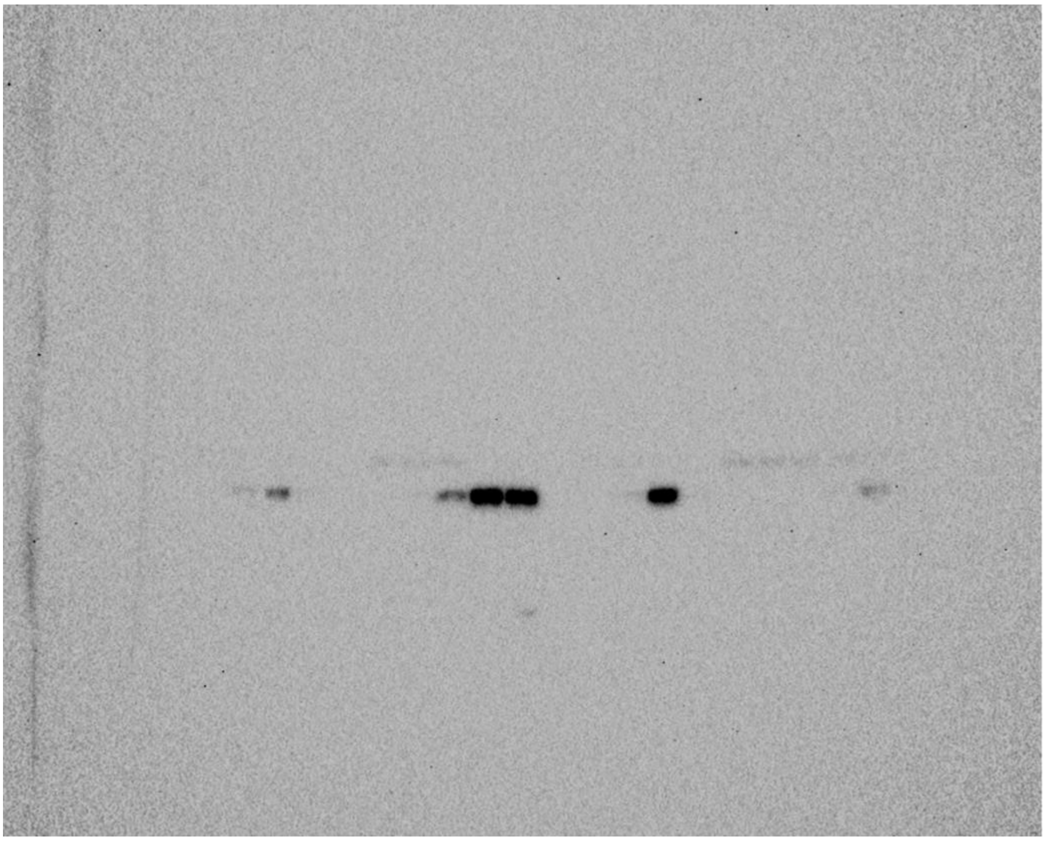

Cleaved Caspase-3

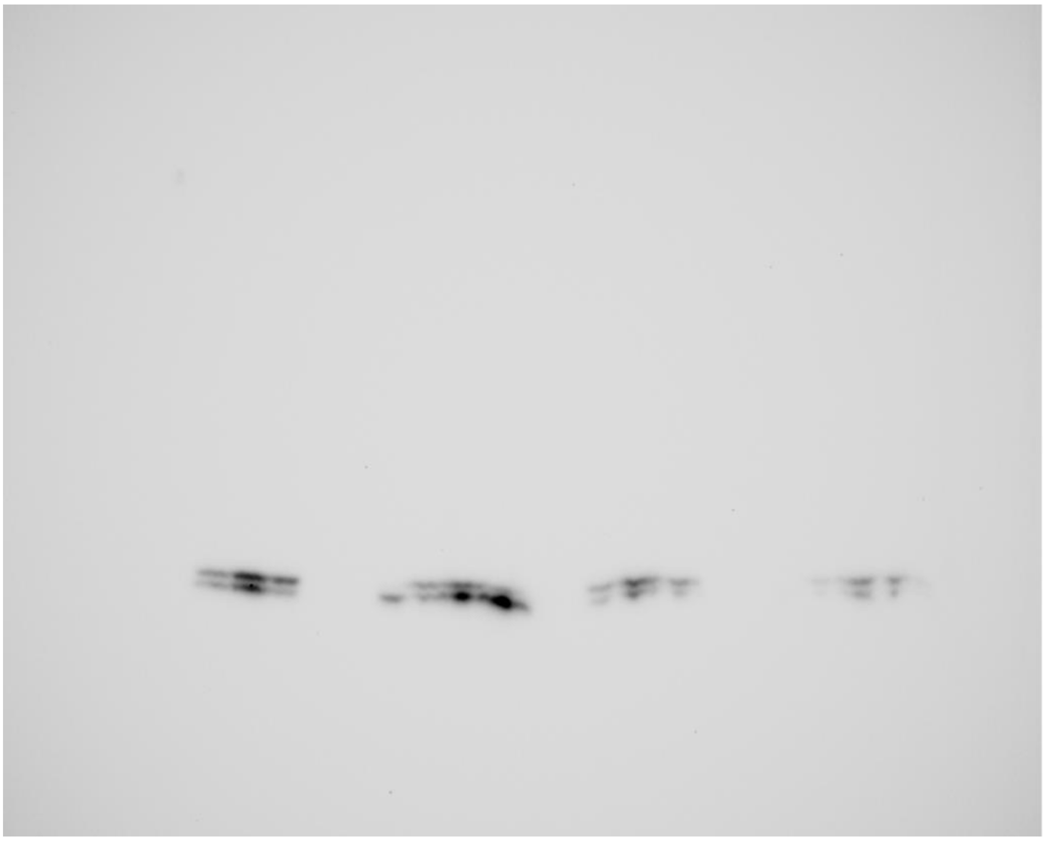

Lelliott et al. Original Westerns Supplementary Figure 3

Actin

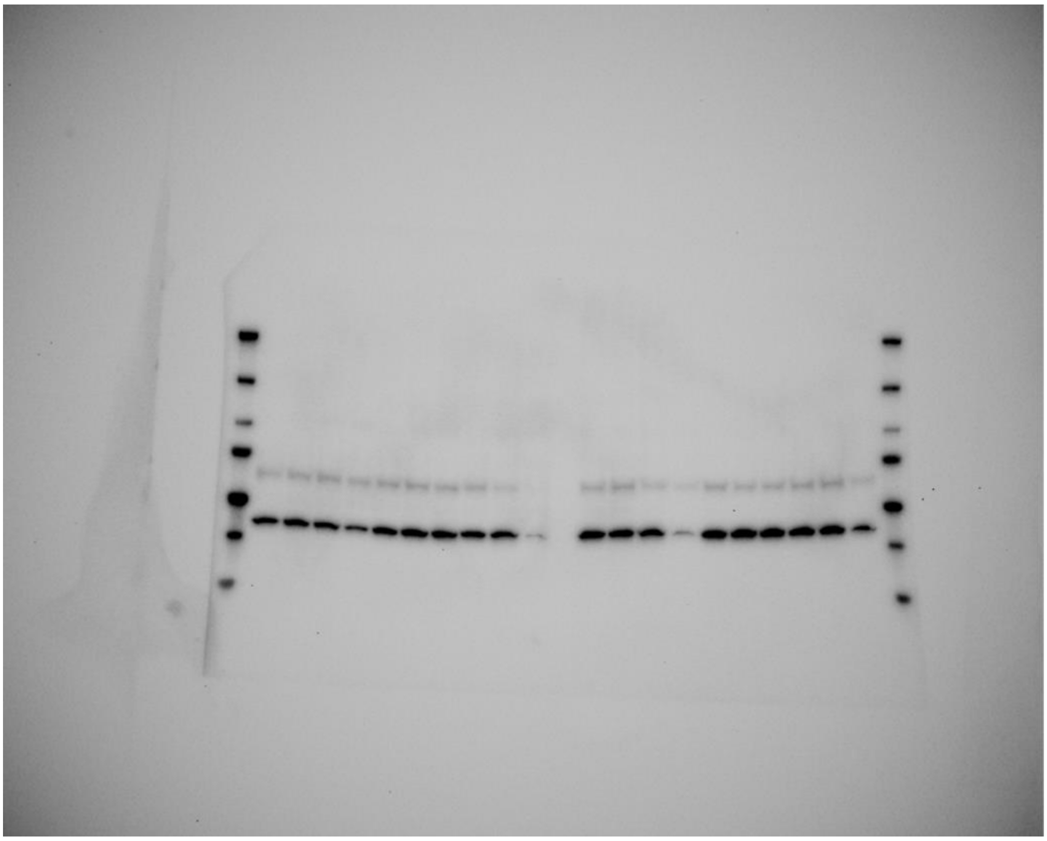

Lelliott et al. Original Westerns Supplementary Figure

Cleaved Caspase 8

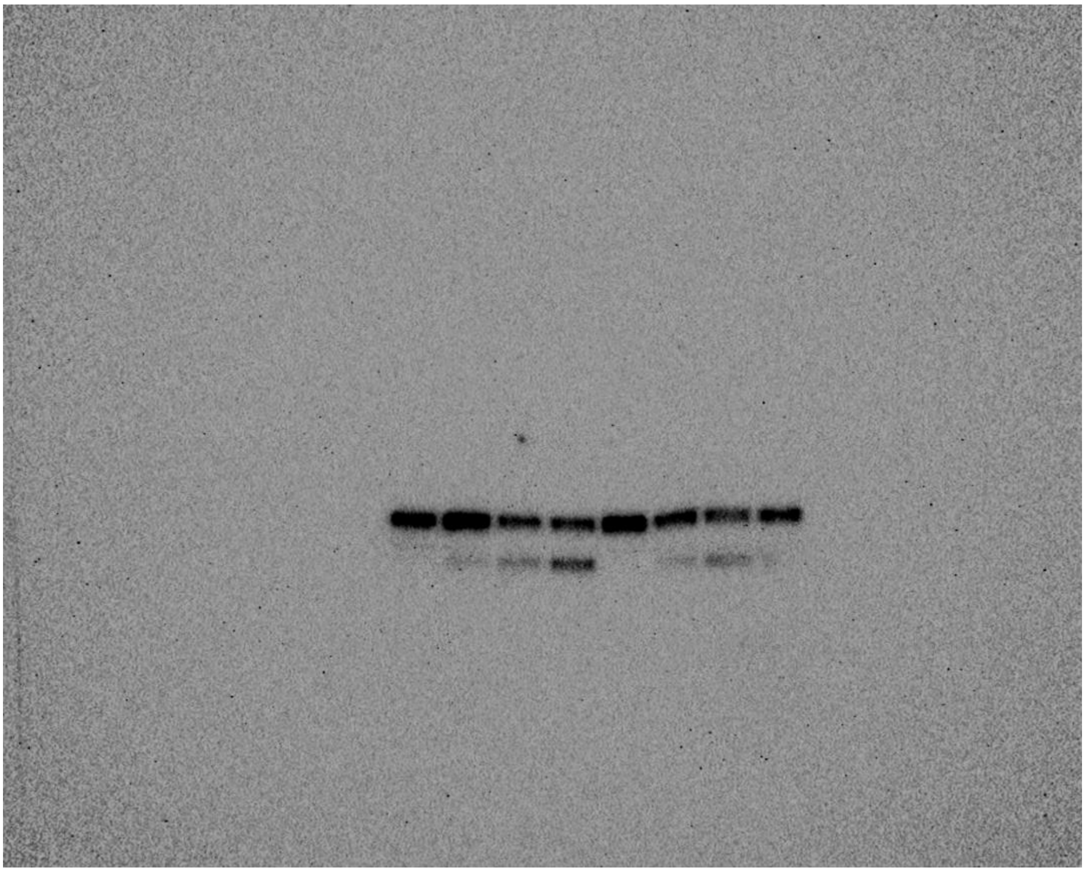

Cleaved Caspase 3

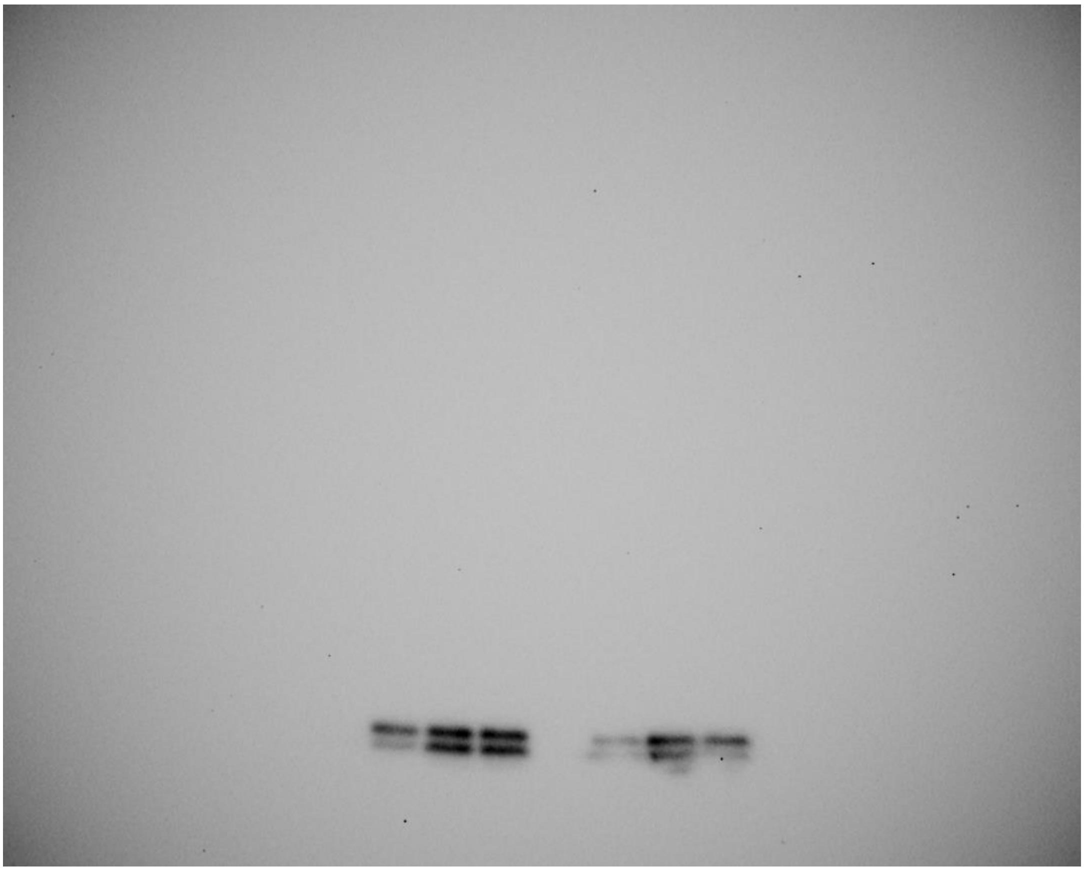

Lelliott et al. Original Westerns Supplementary Figure

XIAP

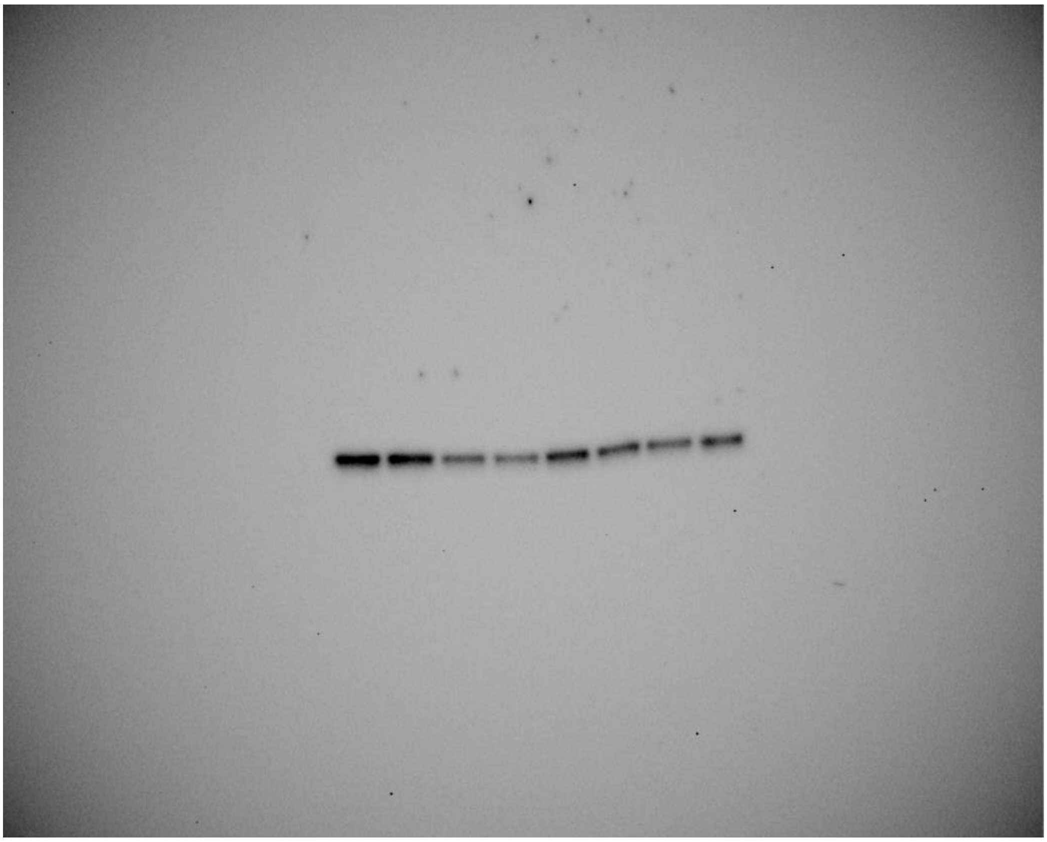

cIAP1

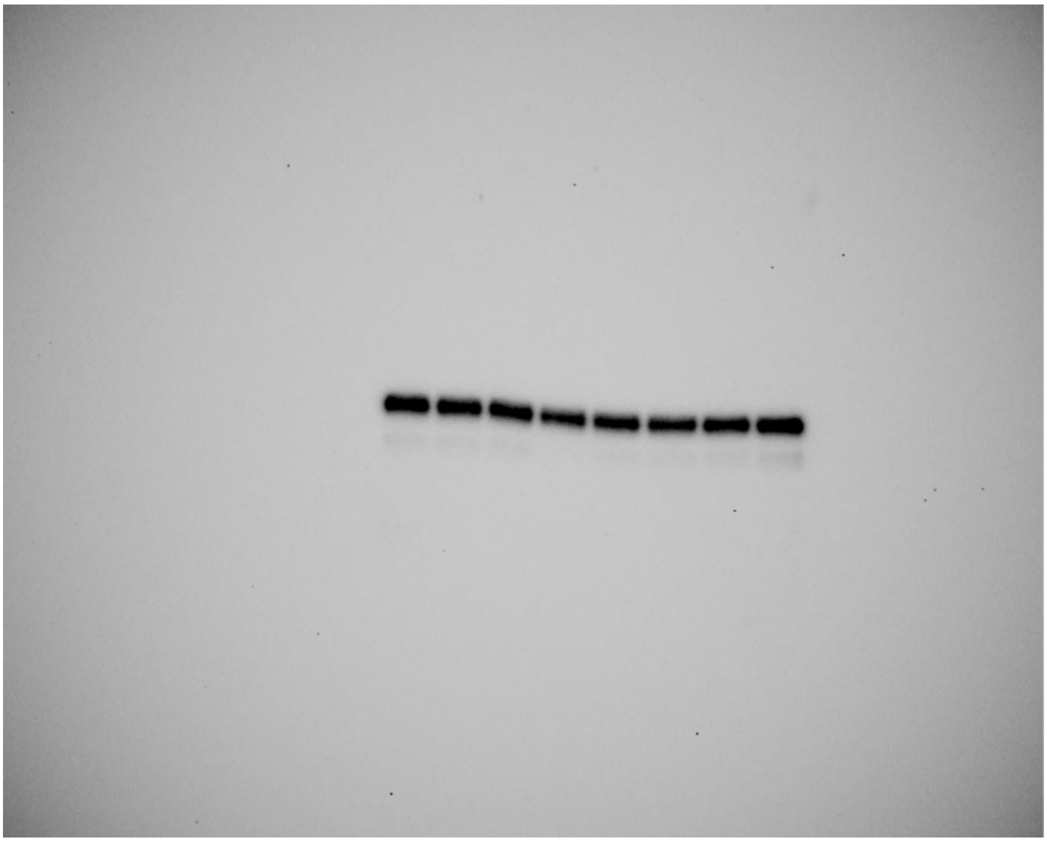

Lelliott et al. Original Westerns Supplementary Figure

Pro-Caspase 8

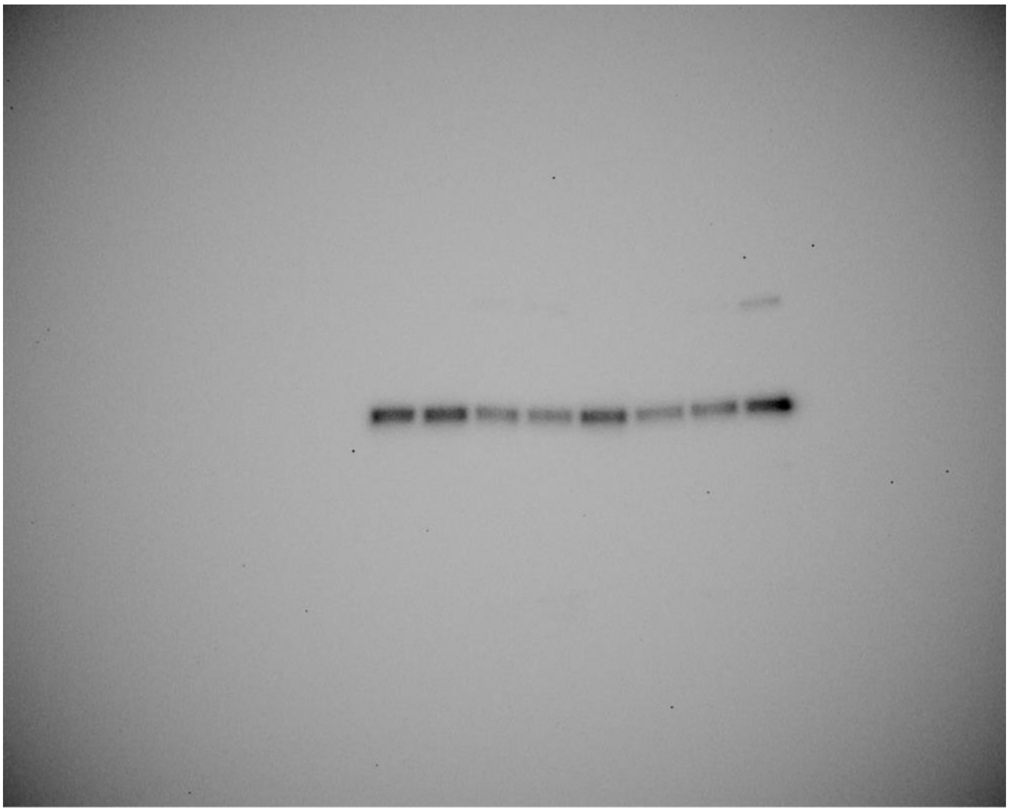

Pro-Caspase 3

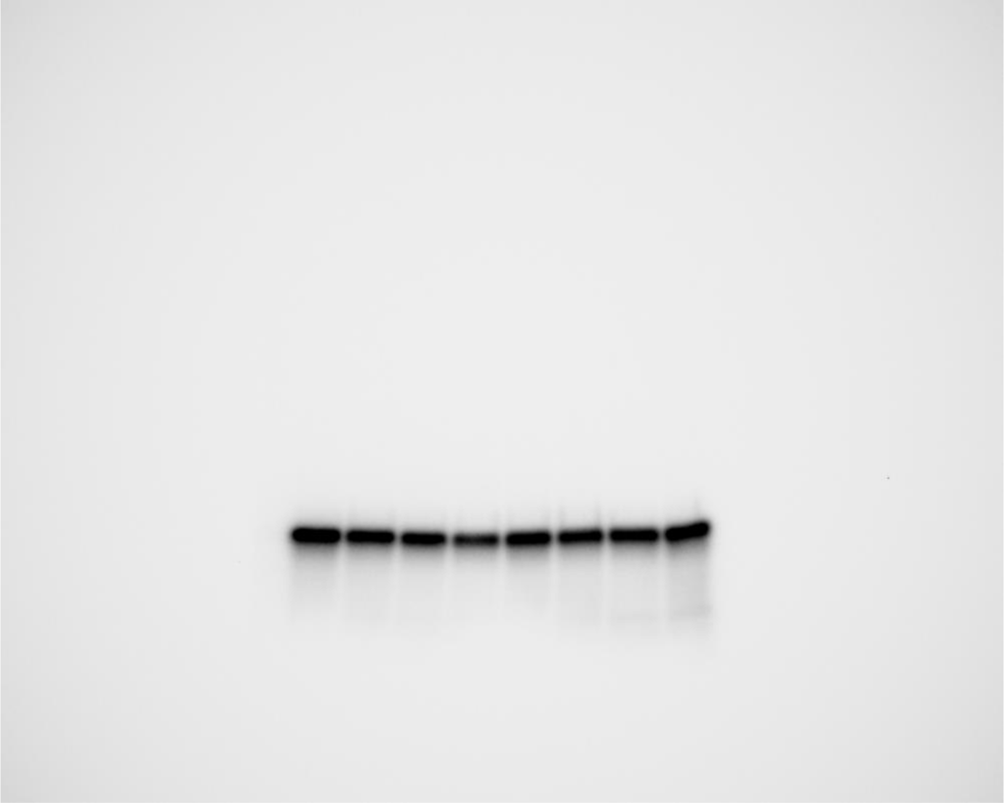

Lelliott et al. Original Westerns Supplementary Figure

Actin

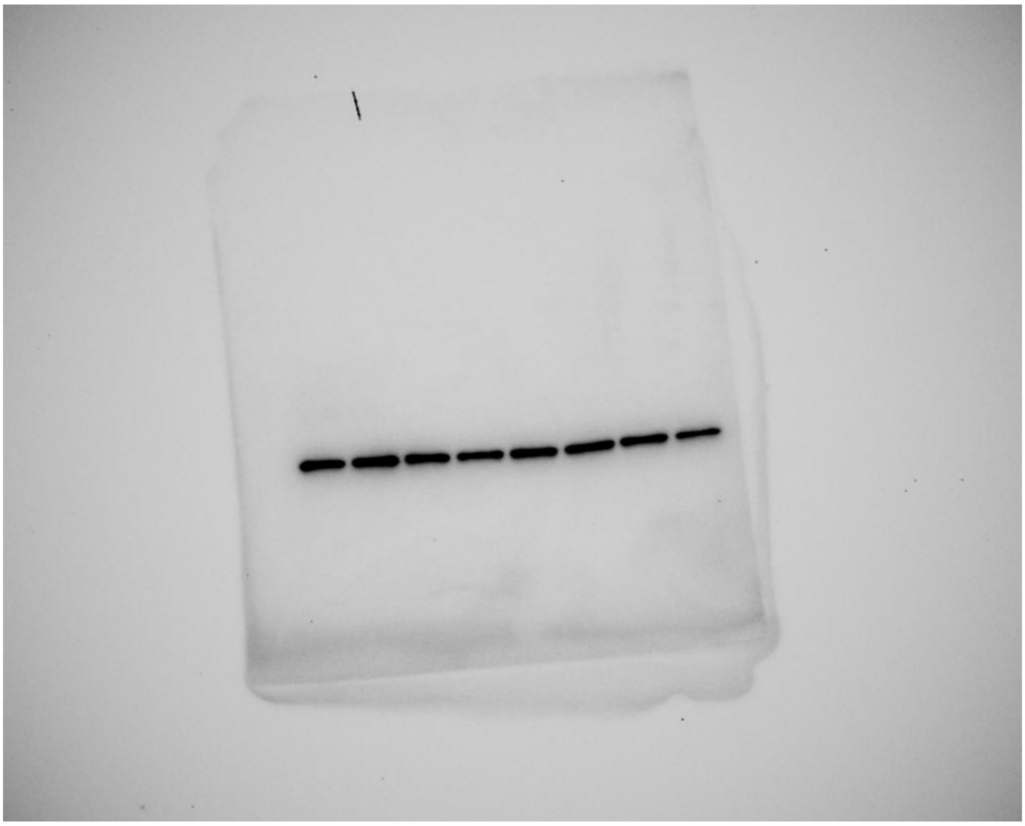

Supplement: Supplementary file 1 — Supplemental Material [file 41418_2024_1369_MOESM1_ESM.pdf]
